# Regenerating zebrafish scales express a subset of evolutionary conserved genes involved in human skeletal disease

**DOI:** 10.1186/s12915-021-01209-8

**Published:** 2022-01-21

**Authors:** Dylan J. M. Bergen, Qiao Tong, Ankit Shukla, Elis Newham, Jan Zethof, Mischa Lundberg, Rebecca Ryan, Scott E. Youlten, Monika Frysz, Peter I. Croucher, Gert Flik, Rebecca J. Richardson, John P. Kemp, Chrissy L. Hammond, Juriaan R. Metz

**Affiliations:** 1 School of Physiology, Pharmacology, and Neuroscience, Faculty of Life Sciences, University of Bristol, Bristol, UK; 2 Musculoskeletal Research Unit, Translational Health Sciences, Bristol Medical School, Faculty of Health Sciences, University of Bristol, Bristol, UK; 3 Institute for Molecular Bioscience, The University of Queensland, Brisbane, Queensland 4072 Australia; 4 Department of Animal Ecology and Physiology, Radboud Institute for Biological and Environmental Sciences, Faculty of Science, Radboud University, Nijmegen, The Netherlands; 5 The University of Queensland Diamantina Institute, The University of Queensland, QLD, Woolloongabba, 4102 Australia; 6 Transformational Bioinformatics, Commonwealth Scientific and Industrial Research Organisation, Sydney, New South Wales Australia; 7 Bone Biology, Garvan Institute of Medical Research, Sydney, New South Wales Australia; 8 St Vincent’s Clinical School, Faculty of Medicine, UNSW Australia, Sydney, New South Wales Australia; 9 Medical Research Council Integrative Epidemiology Unit, Population Health Sciences, Bristol Medical School, University of Bristol, Bristol, UK; 10 School of Biotechnology and Biomolecular Sciences, UNSW Australia, Sydney, New South Wales Australia

**Keywords:** Bone, Collagen, Osteoblast, Musculoskeletal disease, Zebrafish, Scales, Transcriptomics, Extracellular matrix, Ossification

## Abstract

**Background:**

Scales are mineralised exoskeletal structures that are part of the dermal skeleton. Scales have been mostly lost during evolution of terrestrial vertebrates whilst bony fish have retained a mineralised dermal skeleton in the form of fin rays and scales. Each scale is a mineralised collagen plate that is decorated with both matrix-building and resorbing cells. When removed, an ontogenetic scale is quickly replaced following differentiation of the scale pocket-lining cells that regenerate a scale. Processes promoting *de novo* matrix formation and mineralisation initiated during scale regeneration are poorly understood. Therefore, we performed transcriptomic analysis to determine gene networks and their pathways involved in dermal scale regeneration.

**Results:**

We defined the transcriptomic profiles of ontogenetic and regenerating scales of zebrafish and identified 604 differentially expressed genes (DEGs). These were enriched for extracellular matrix, ossification, and cell adhesion pathways, but not in enamel or dentin formation processes indicating that scales are reminiscent to bone. Hypergeometric tests involving monogenetic skeletal disorders showed that DEGs were strongly enriched for human orthologues that are mutated in low bone mass and abnormal bone mineralisation diseases (*P*< 2× 10^−3^). The DEGs were also enriched for human orthologues associated with polygenetic skeletal traits, including height (*P*< 6× 10^−4^), and estimated bone mineral density (eBMD, *P*< 2× 10^−5^). Zebrafish mutants of two human orthologues that were robustly associated with height (*COL11A2*, *P*=6× 10^−24^) or eBMD (*SPP1*, *P*=6× 10^−20^) showed both exo- and endo- skeletal abnormalities as predicted by our genetic association analyses; *col11a2*^*Y228X/Y228X*^ mutants showed exoskeletal and endoskeletal features consistent with abnormal growth, whereas *spp1*^*P160X/P160X*^ mutants predominantly showed mineralisation defects.

**Conclusion:**

We show that scales have a strong osteogenic expression profile comparable to other elements of the dermal skeleton, enriched in genes that favour collagen matrix growth. Despite the many differences between scale and endoskeletal developmental processes, we also show that zebrafish scales express an evolutionarily conserved sub-population of genes that are relevant to human skeletal disease.

**Supplementary Information:**

The online version contains supplementary material available at 10.1186/s12915-021-01209-8.

## Background

The vertebrate skeleton is composed of non-mineralised cartilage and mineralised bone, enamel, and dentine structures. Skeletal structures have a collagen-rich matrix that provides shape, organ protection, endocrine, and locomotive functions to the organism. Bone is formed by evolutionarily conserved processes via either endochondral or intramembranous (dermal) ossification. Endochondral bone formation occurs through progressive remodelling of a cartilage template into bone; intramembranous bone formation occurs by mesenchymal condensation of osteoblasts. Bones are dynamic tissues and the formation, maintenance and remodelling of the calcified extracellular matrix (ECM) are controlled via a tight coupling between the functional activity of the bone-building osteoblasts and bone-degrading osteoclasts [1]. In most vertebrates, osteoblasts can become entrapped within the bone matrix and differentiate into osteocytes forming a lacunocanalicular network [2]. A fundamental difference between tetrapods and most teleost fish is that bone is devoid of osteocytes in advanced (neo-)teleosts, including the medaka [3]. In zebrafish, most bony elements contain osteocytes while some do not. As osteocytes act as mechanosensors, recent studies showed that both teleost species dynamically express osteocyte marker sclerostin (*sost*) at sites of loading [4]. Zebrafish show a bone remodelling response depending on the degree of exercise-induced loading of bones [5, 6]. Osteoblasts therefore have potential to fulfil a mechano-sensing role to initiate functional coupling to osteoclasts in the bone remodelling cycle and that this can occur without or with reduced osteocyte function.

While skeletal regenerative capacity in mammals is limited to remodelling and injury healing, teleost exoskeletal structures composed of fin rays and elasmoid scales can undergo epimorphic regeneration and subsequent rapid regrowth of a mineralised tissue [7]. This process can therefore offer a potential route to identify new pathways and targets involving calcified matrix formation. Zebrafish are increasingly used as model systems for musculoskeletal (MSK) research, due to their genetic tractability, availability of transgenic reporter lines, and the dynamic imaging opportunities they offer; and because, while there are some differences, the molecular processes underpinning bone formation are comparable with those of terrestrial vertebrates [8]. In recent years, zebrafish fin regeneration has been extensively studied and has revealed many of the mechanisms that underpin blastema formation, and skeletal dedifferentiation and re-differentiation during tissue regrowth [9].

The elasmoid scales of zebrafish are exoskeletal ectodermal appendages, part of the dermal skeleton, which function as a protective armour and a calcium reservoir [10, 11]. They have two layers: a calcified layer on the exterior and a type I collagen fibrillary plate to the inner side. Whilst lacking osteocytes, scales are decorated with matrix-building cells lining the internal (hyposquamal) and calcified external layer. The nomenclature involving matrix-building cells is diverse and they have interchangeably been called ameloblasts, elasmoblasts, scleroblasts, and osteoblasts [10, 12–15]. Developmental, molecular and genetic studies have helped establish that elasmoid scales, like other elements of the dermal skeleton, likely derive from ancestral odontodes [16, 17]. In fact, in the vertebrate common ancestor, the earliest skeletal tissues to biomineralise were likely to be part of protruding exoskeletal structures from the epidermis that provided protection to the organism. Exoskeletal elements have been strongly reduced or completely lost in terrestrial animals during evolution but have been retained in bony fish [18]. In contrast to lower jaw endochondral bones which are derived from the ectodermal neural crest, dermal scales are formed by mesoderm-derived mesenchymal precursor condensations and not (ectodermal) trunk neural crest cells during zebrafish development [17]. The subsequent epidermal placode (or papilla) formation is under spatiotemporal control of hedgehog, Wnt/β-catenin, Fgf, and Eda signalling pathways [19]. In common with intramembranous flat bones, the scales are then formed and mineralised directly by *de novo* differentiated *sp7*^*+*^ cells [20, 21]. On an ontogenetic scale, *sp7*^*+*^ cells are located at the hyposquamal side of the plate.

Natural shedding or mechanical removal of a scale initiates a wound healing phase followed by an epimorphic regeneration process and has recently gained attention in studies aiming at a deeper understanding of mechanisms of calcified tissue growth, regeneration, and repair. The regeneration phase leads to the formation of a small mineralised scale as early as two days post-harvest (dph) [15, 22, 23]. Expression of early osteoblast marker *sp7* is detected and increases during growth of the scale, that is followed by increased expression of mineralisation marker *entpd5a* but also Wnt inhibitor and osteocyte marker *sost* [24]. Regenerating scales have a high density of ECM-secreting cells at the posterior edge of the plate forming the leading growth plane whose dynamics can be tracked *in toto* [12]. These cells form a monolayer and provide conditions that favour precipitation of apatites, such as hydroxyapatite, into a type I collagen-rich matrix. The hyper-mineralised layer on the exterior side can be resorbed by tartrate-resistant acid phosphatase (TRAP)-positive osteoclasts [25, 26]. Similar to endoskeletal bones in fish and terrestrial animals, scale matrix turnover responds to exposure of prednisolone, alendronate, chronic hyperglycaemia, and fatty acids (e.g. OMEGA-6) highlighting evolutionarily conserved mechanisms of calcified matrix metabolism [24, 27–29]. Moreover, the collagen matrix of scales is organised in a woven plywood manner resembling similarities to lamellar bone [30–32] and show a response to mechanical injury (performed *in vivo*) with recruitment of *trapc-*expressing cells to the site of damage [33].

All those features warrant a deeper analysis of gene expression and the pathways involved in dermal scale regeneration. In this study, we describe the transcriptome of ontogenetic and regenerating scales and show that scales have a strong osteogenic expression profile. We also show that differentially expressed genes (DEGs) are enriched for genes associated with different modes of bone formation and human MSK disease.

## Results

### The transcriptome of ontogenetic and regenerating scales contains bone growth and matrix genes

**Fig. 1:**
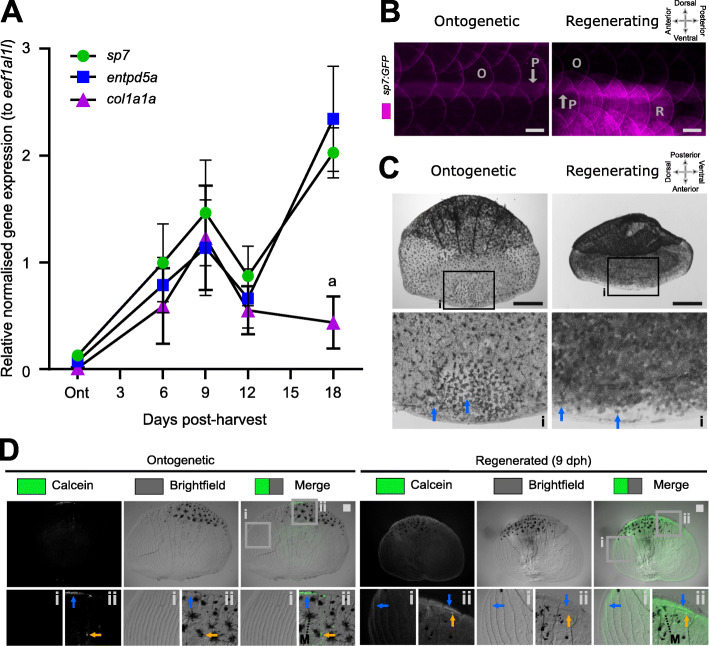
Regenerating scales express metabolic active bone growth markers at 9 days post-harvest. **A** Quantitative real-time PCR of osteoblast (*sp7*), mineralisation (*entpd5a*), and matrix growth (*col1a1a*) amplicon expression (*n* = 4 fish). Two-way ANOVA analysis showed that there was no interaction factor variance (*p* = 0.133) but that time (*f*(4) = 9.47, *p*< 0.001) and amplicon (*f*(2) = 4.62, *p*< 0.05) had independent statistically significant effects. Tukey’s multiple comparison determined that *col1a1a* was significantly lower expressed at 18 days post-harvest (dph) compared to other amplicons (a). **B** *In toto* fluorescent stereomicroscope images of flanks from *sp7:GFP* transgenic fish showing ontogenetic (O) and 9 dph regenerated scales (R) in an anterior (left) to posterior (right) direction). Note the auto-fluorescent pigment stripe (P) and the compass orientation is used to depict *in toto* scales in this paper with the distal (posterior) edge pointing caudally. **C** *In situ* alkaline phosphatase staining of a pre-collection and 9 dph scale with insets (i) showing anterior part of the scale. Blue arrows indicate ALP+ cells which appear to be larger and more extended in ontogenetic than regenerating scales. Compass orientation is used to depict *in situ* scales in this paper as the anterior region was closest to the scale dermal socket (pre-harvest). **D** Representative stereomicroscope images of live calcein green staining labelling newly deposited calcium phosphate in ontogenetic and regenerating scales (*n* = 4 fish each condition). Blue arrow indicates elevated levels of calcein green labelling compared to surrounding signal whereas orange arrows indicate small puncta of enhanced signal. Insets show lateral circuli (i) and posterior epidermal (containing pigmentating melanocytes (M) region (ii). Scale bar: 100 μm

We aimed to define the transcriptome during peak growth of the regenerating scale. Regenerating scales showed a temporal increase of matrix growth and calcification markers (*sp7*, *entpd5a*, and *col1a1a*) and we observed that this peaked around 9 dph compared to original scales formed during development (ontogenetic) (Fig. 1A). At this time point, the number of *sp7* positive cells were visually increased (Fig. 1B) combined with an increased alkaline phosphatase (ALP) activity (Fig. 1C) and calcein green labelling (Fig. 1D), similar to previous reports [12, 24]. To identify the genes involved in calcified matrix growth during the regeneration process, we performed RNA-sequencing (RNA-seq) on ontogenetic and regenerating scales 9 days into their regeneration.

**Fig. 2:**
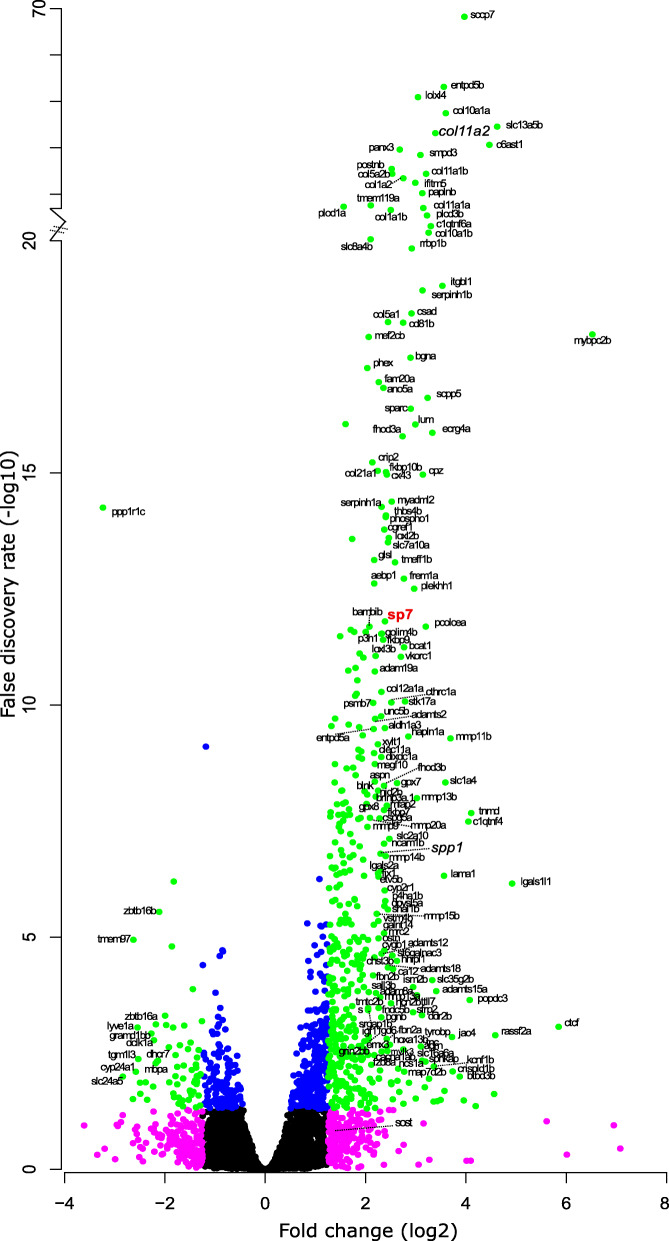
Volcano plot of RNA-seq data comparing expression between ontogenetic and 9 days post-harvest regenerating scales. Relative fold change (log_2_) and false discovery rate (-log_10_ converted) of coding RNA sequences that were expressed in both ontogenetic and regenerating scales. Green (≥±1.25 fold change, ≥ 1.3 FDR), magenta (≥±1.25 fold change, < 1.3 FDR), blue (<±1.25 fold change, ≥1.3 FDR), and black (not passing any threshold) coloured dots mark the different criteria

**Fig. 3:**
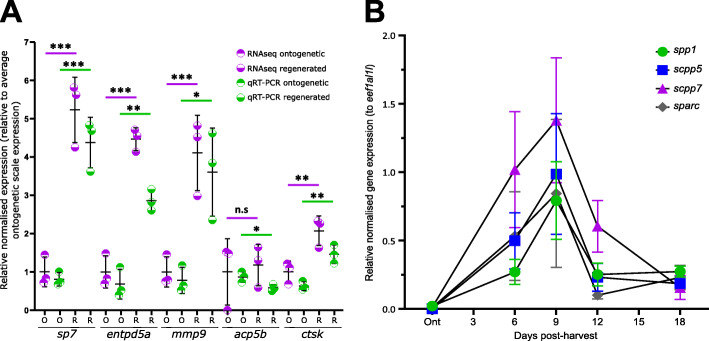
Validation of RNA-sequencing dataset and SCPP repertoire gene expression during scale regeneration. **A** Comparison of relative expression levels of qRT-PCR (unpaired *t* test) and transcriptomic analysis (false discovery rate) of selected amplicons. Total RNA used for RNA-seq experiment was used. **B** Quantitative real-time PCR (qRT-PCR) of secretory calcium-binding protein (SCPP) family genes that were differentially expressed in the RNA-seq dataset (*n* = 4 fish). Two-way ANOVA analysis showed that there was no interaction factor variance (*p* = 0.882) but that time (*f*(4) = 11.05, *p*=0.001) was the only independent statistically significant effect on amplicon expression

A total of 13,170 protein coding genes were consistently expressed (i.e. 51.4% of total protein coding genome) in both ontogenetic and regenerating scales (Additional file 1). Principal component analysis (PCA) of the two groups of all genes expressed showed that ontogenetic and regenerating scale groups cluster together (Additional file 2: Fig. S1A) and that there was a high level of correlation (Pearson’s correlation of 97.1%, Additional file 2: Fig. S1B), confirming similar expression patterns within the PCA groups. Setting the arbitrary threshold at 1.25 log_2_ fold change and a false discovery rate (FDR) of < 0.05 showed that 604 protein coding genes had substantial differences in gene expression (Additional file 3). We observed a skew towards upregulation of genes (*n* = 514 compared to *n* = 90 downregulated) (Fig. 2); consistent with regenerating tissues such as the caudal fin [34, 35]. The two top DEGs that were highly downregulated were also teleost specific, and a homology search revealed that both genes encoded different proteins [i.e. ENSDARG00000068621 (*si:ch211-181d7.3*) and ENSDARG00000088274 (*si:ch211-181d7.1*)] (Additional file 2: Fig. S2). Both genes have NOD-like receptor (NLR) domains that possess NACHT (NTPase) and leucine-rich repeat (LRR) protein domains, important for innate immune system antigen interactions [36]. To validate the RNA-seq dataset, quantitative real-time PCR (qRT-PCR) was performed on mineralised tissue growth and degradation markers and showed concordant data between RNA-seq and qRT-PCR assays of all selected amplicons (Fig. 3A).

Among the top DEGs, we noticed a high number of collagen and cell adhesion-related genes, but interestingly also *secretory calcium-binding phosphoprotein* (*scpp*) *5* and *scpp7* genes (Table 1). These two *scpp* genes are teleost specific that belong to the *SCCP* family of genes. In vertebrates, this so-called *SCPP* repertoire of genes tends to group together in the genome as two main clusters. During evolution, these two clusters developed high variability between species as a result of a high rate of gene gains and losses but are derived from two *SCPP* precursors (*SPARC* and *SPARCL1*) that originated together with the advent of biomineralisation in a vertebrate common ancestor [37]. In humans, the two clusters on chromosome 4 harbour genes that are predominantly expressed in either (hypermineralised) enamel matrix (*AMBN*, *ENAM*, *AMTN* and *ODAM*) or in bone and dentin matrix (*secreted phosphoprotein 1* (*SPP1*, commonly known as Osteopontin (opn)), *DSPP*, *DMP1*, *IBSP* and *MEPE*). In zebrafish, both *scpp* clusters have orthologues that show a form of genomic clustering. The zebrafish equivalent of ‘enamel’ cluster (chromosome 1) lacks an *AMTN* orthologue but contains *scpp5*, *scpp7* and *scpp9* genes in this region*,* whilst the orthologous ‘bone/dentin’ cluster (chromosome 10) only harbours an *SPP1* orthologue and a teleost annotated *scpp8* gene. Both *sparc* and *sparcl1* ancestral *scpp* repertoire genes are also present in the zebrafish genome. As elasmoidin matrix is composed of a lamellar matrix resembling similarity to enamel, we wondered whether the ‘enamel’ or ‘bone/dentin’ *scpp* repertoire gene clusters were differentially expressed in regenerating scales. A look up of the gene expression of all zebrafish orthologues including zebrafish specific *scpp* genes showed that the ‘enamel’ cluster, *ambn*, *enam* and *odam* were not or very lowly expressed whilst teleost specific *scpp5* and *scpp7* were differentially upregulated (Table 2). The ‘bone/dentin’ cluster *spp1* gene was substantially upregulated as was ancestral *SCPP sparc* (osteonectin (osn)) that is also associated with bone development (Table 2). We next assessed the temporal expression of DEG *SCPP* genes (*spp1*, *scpp5*, *scpp7* and *sparc*) during scale regeneration. This showed that these four genes peaked their expression at 9 dph (Fig. 3B), and this pattern was similar to *col1a1a* and not *sp7* or *entpd5a* (Fig. 1A). These observations indicate that the elasmoid scale growth relies on ‘bone/dentin’ type of *scpp* repertoire gene expression rather than ‘enamel’ cluster genes. Scale growth therefore resembles an osteoanabolic process.

**Table 1:**
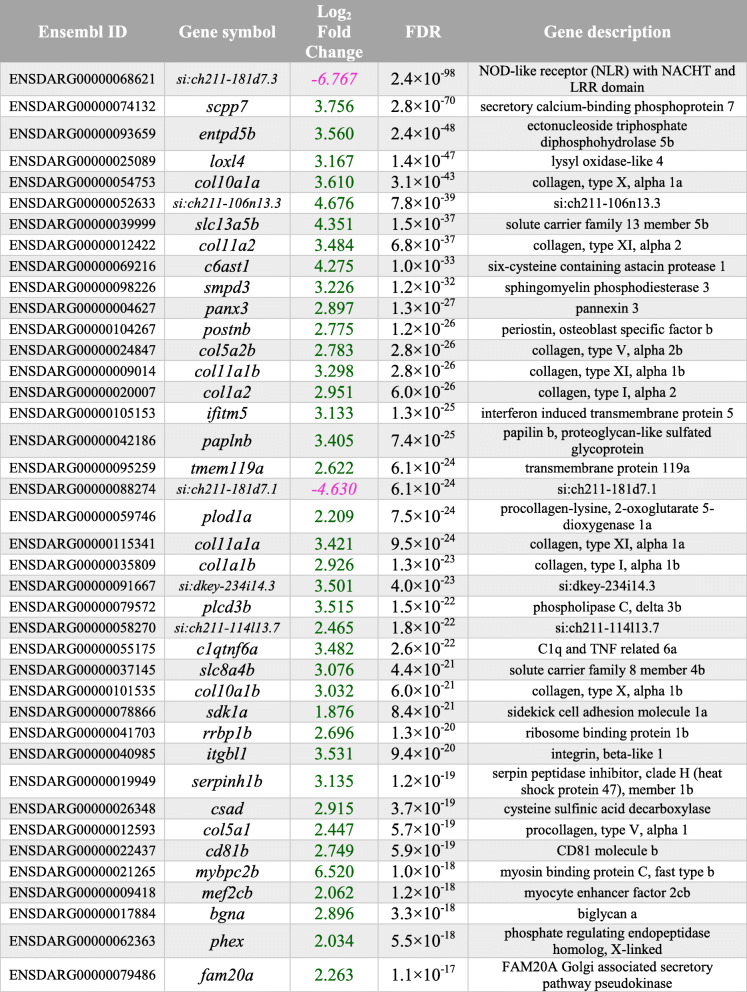
Top-40 smallest differentially regulated transcripts. Thirty-eight out of 40 of the top-40 differentially expressed genes are up (green) regulated, whilst only two were down (magenta) regulated. Log_2_ fold change in regenerated versus ontogenetic scales with false discovery rate (FDR) are shown. Top-40 was selected based on the lowest FDR numerical

**Table 2:**
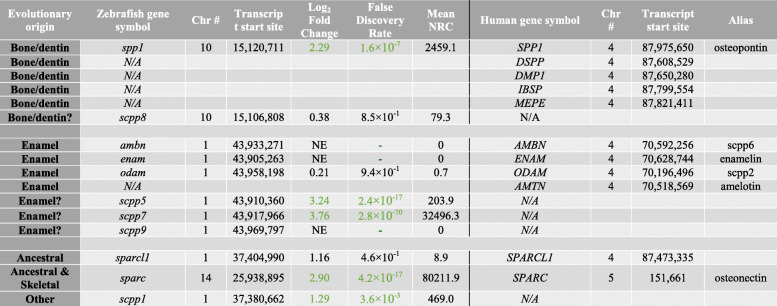
Differential expression of scpp gene clusters in scales corresponding with human orthologue SCPP clusters associated with site-specific expression

SCPP cluster genes associated with enamel formation are barely expressed in the RNA-seq dataset. Abbreviations: *N/A* not annotated, *NE* not expressed, *NRC* normalised read count; ?: suggestive expression based on genomic clustering in zebrafish. Table separates human SCPP ‘bone/dentin’ genomic cluster, SCPP ‘enamel’ genomic cluster, and human ‘ancestral’ SCPP genes. Green numbering represents passing arbitrary Log_2_ fold change thresholds. Mean NRC is mean read count taken from the three ontogenetic and regenerating samples. AMELX and AMELY have been omitted from analysis as zebrafish do not have sex chromosomes

Indeed, amongst the DEGs we observed osteoblast factors such as *sp7*, *entpd5a/b*, *postnb*, *yap1*, *smpd3*, *phospho1*, *plod1a*, *plod2* and *plod3*; genes related to ECM growth, collagens (predominantly fibrillar collagens) and collagen remodelling including *bmp1a*, *mmp9*, *spp1*, *sparc*, *col1a1a/b*, *col1a2*, *col5a1, col5a2a/b, col10a1a/b*, *bgna/b*, *col11a1a/b* and *col11a2* [38]. We also observed elevated expression of genes normally associated with endochondral ossification: *col11a1a/b* and *col11a2*, *ihha*, *ptch2*, *dlx5a*, *ostn* (osteocrin) and *mef2cb*, suggesting that scale DEGs have a relationship with both modes of ossification [39]. Osteoclast and monocyte-related factors (e.g. *tnfrsf11a* (*RANK*), chloride channel *clc7n7*, *cathepsin K* (*ctsk*) and *acp5a/b (trapc*)) but also markers associated with osteocyte differentiation; *sost*, *wnt3a, lrp4, lrp5* and *dkk1a* were expressed in both ontogenetic and regenerating scales, but differential expression was not observed (Additional file 1). These results indicate osteoblast genes are elevated to a greater degree than those associated with osteoclasts concordant with an osteoanabolic process.

### The collagen processing pathway is highly enriched and upregulated during scale regeneration

**Fig. 4:**
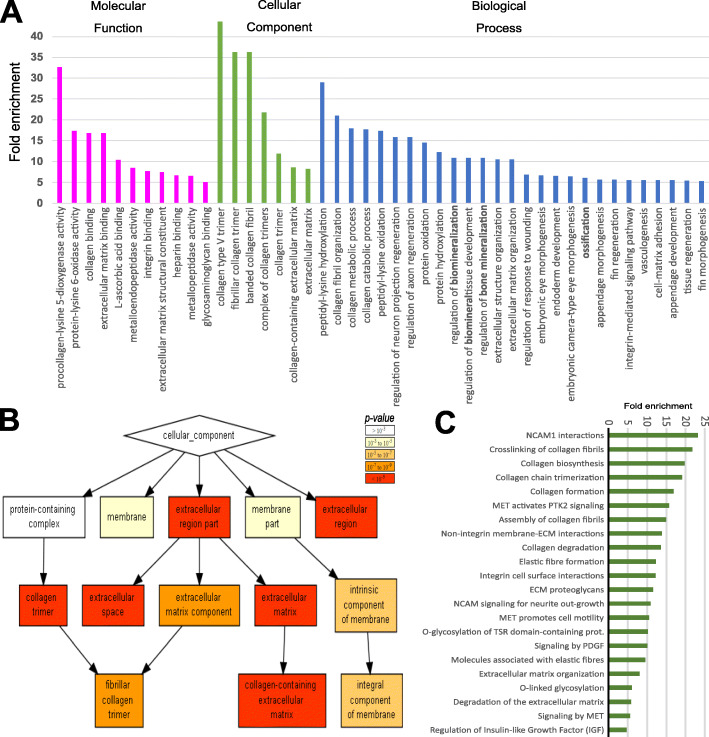
Gene ontology and pathway analysis. **A** Panther gene ontology (GO) analysis showing high enrichment (> 5 fold) of collagen, ossification, and ECM related terms. **B** GOrilla software showing cellular component hierarchical clustering of collagen-related GO terms. **C)** Graphical representation of enrichment (> 5 fold) of differentially expressed gene set using Panther Reactome Pathway overrepresentation test (v65) that showed enrichment of cell adhesion, collagen matrix and processing and glycosylation terms

To identify biological pathways overrepresented among DEGs, we performed gene ontology (GO) enrichment analysis. We performed PANTHER GO analysis and in accordance with a regenerating growing tissue there were many GO terms that included ‘morphogenesis’, ‘development’, ‘regeneration’ and ‘collagen’ in their descriptions (Fig. 4A and Additional file 4). There were also bone-specific GO terms such as ‘regulation of bone biomineralization’ (GO:0110149; 10.9-fold, FDR=0.018), ‘ossification’ (GO:0001503; 6.0-fold, FDR=0.0019), and ‘skeletal system development’ (GO:0001501; 3.6-fold, FDR=5.3 × 10^-6^). Additional bone matrix associated processes such as ‘calcium-ion binding’, ‘glycosaminoglycan binding’ and ‘matrix metalloprotease (MMP) activity’ were identified (Additional file 2: Fig. S3 and S4). A separate GOrilla GO analysis broadly confirmed these findings and visualised the GO terms by hierarchical clustering (Fig. 4B; Additional file 2: Fig. S3 and S4; Additional file 5).

**Fig. 5:**
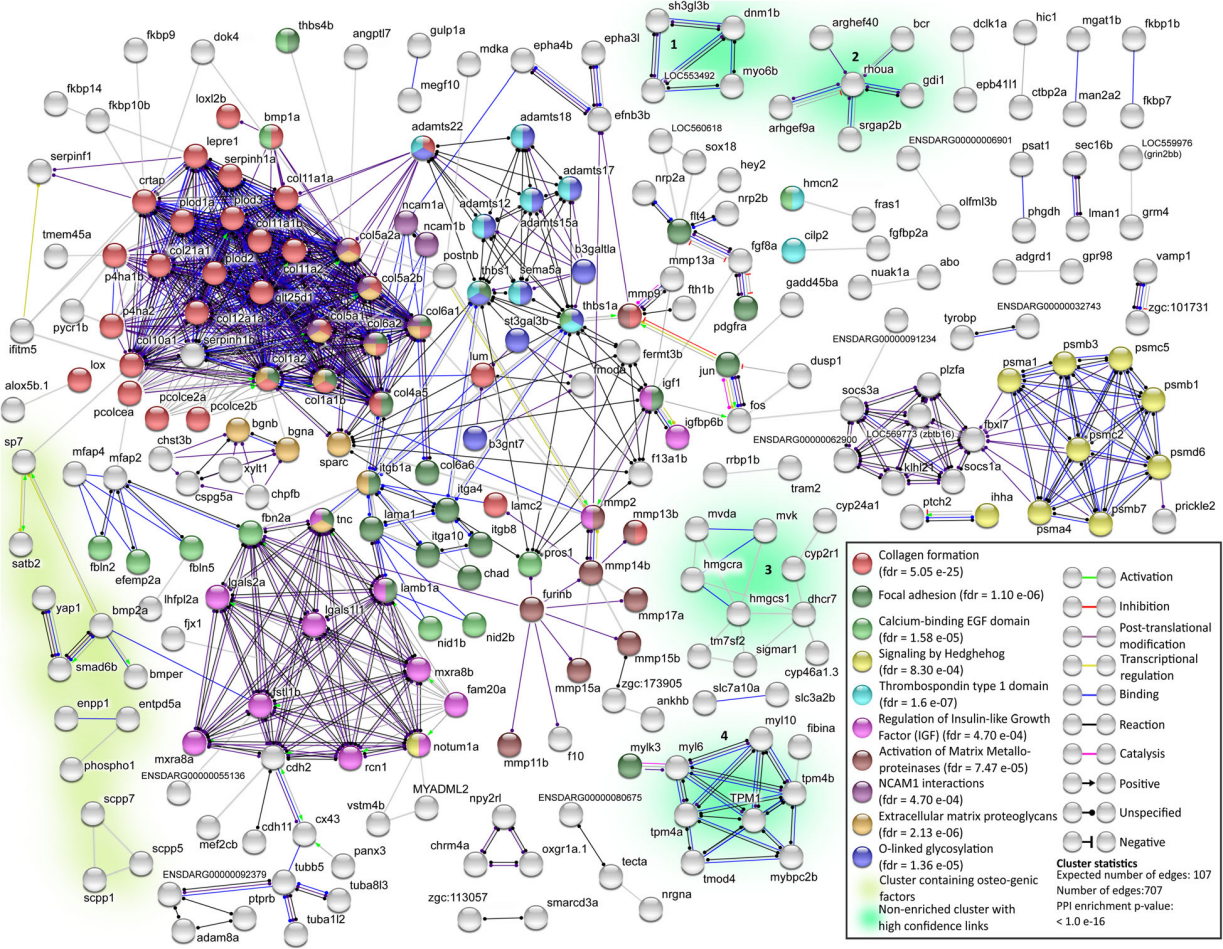
STRING network analysis of DEGs. Only DEG proteins with 1 or more protein-protein interactions (PPI) (indicated as edges) were shown

We also observed cell adhesion-related terms and PANTHER ‘Pathways’ showed that the ‘integrin signalling pathway’ (P00034; 3.5-fold, FDR=0.016) was the only enriched signalling pathway, which is known to bind collagen during cell adhesion-related processes [40] (Additional file 5). To further identify potential clusters of genes, PANTHER ‘Reactome pathways’ protein-protein interactions (PPI) analysis was used. This showed consistent enrichment of players involved in collagen matrix biosynthesis (e.g. ‘O-linked glycosylation’ and ‘ECM proteoglycans’) but also ECM interactors such as neural cell adhesion molecule (NCAM) and insulin-like growth factor (IGF) (Fig. 4C and Additional file 6). STRING Network analysis showed that the DEGs PPI network has 6.6-fold higher number of nodes (connections) compared to the whole reactome (*P*< 1.0 × 10^-16^). There were ten clusters with distinct functions that were significantly enriched that were highly associated with collagen-rich ECM and cell adhesion, but also with IGF and hedgehog signalling (Fig. 5). Note that STRING added 10 ‘high-scoring’ interactors to the network that were not DEGs (Additional file 2: Fig. S5A). Additional four unlinked clusters were related to cytoskeletal structure or cell motility (Fig. 5 and Additional file 2: Table S1). Allowing less stringent interaction evidence scores (≥ 0.4) showed that *sp7* and *spp1* factors have connections with both the collagen and bone factor clusters, the latter showing connections to adjacent osteogenic clusters that for example contain *phospo1* and *ostn*, but also to *sparc*, *scpp5*, *scpp7* and *col10a1a* (Additional file 2: Fig. S5B). Together, the GO and PPI analyses indicated that DEGs were enriched for biological pathways involved in bone formation and collagen-matrix synthesis, consistent with increased osteoblast activity in a regenerating tissue.

**Fig. 6:**
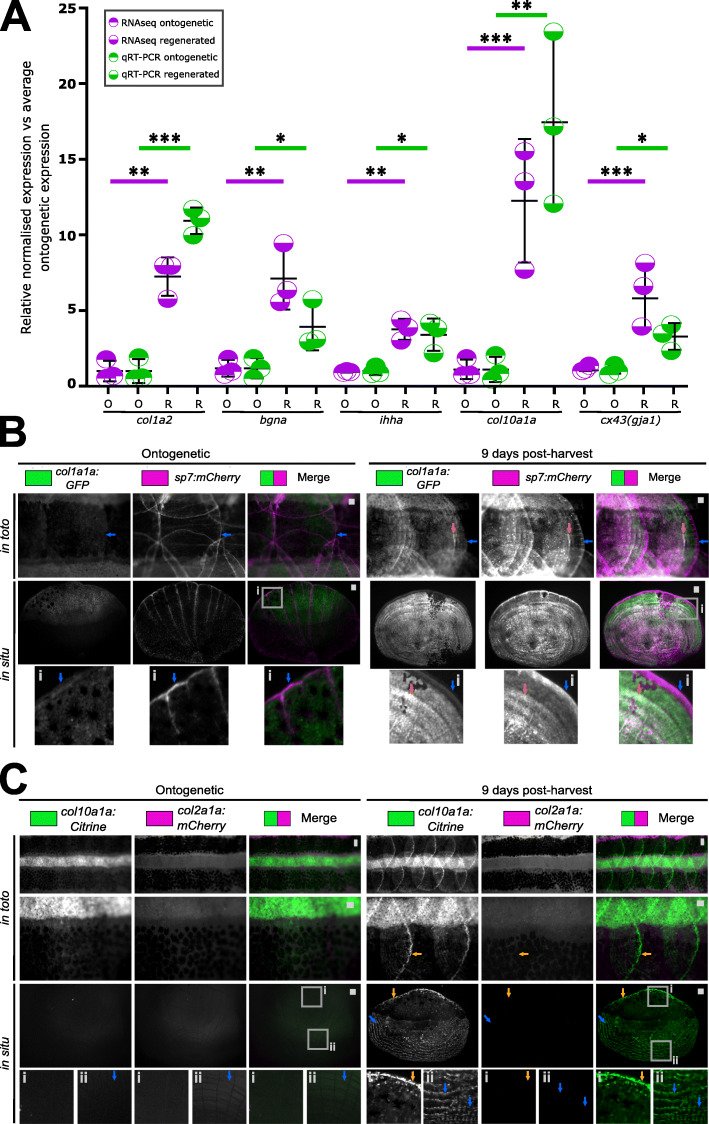
Expression validation of gene ontology findings. **A** Relative expression levels of qRT-PCR (unpaired *t* test) and transcriptomic analysis of amplicons (false discovery rate) found in the collagen, proteoglycan and hedgehog signalling networks. **B** Images of *in toto* and harvested scales (*in situ*) of *col1a1a:GFP* and *sp7:mCherry-NTR* double transgenic fish (*n* = 4 each condition). Blue arrow indicates the posterior (distal) fringe with high expression of mCherry. Orange arrow points at co-expression of GFP and mCherry. **C** *In toto* and *in situ* stereomicroscope images of *col10a1a:Citrine* and *col2a1a:mCherry* double transgenic ontogenetic and regenerating scales (*n* = 4 fish each condition). Orange arrows indicate Citrine signal at the posterior distal edge (inset i) while blue arrow points at a newly forming lateral circulus (inset ii) of the scale. Scale bar: 100 μm

We next verified RNA expression of *col1a2,* osteoblast deposited collagen *col10a1a,* the collagen nucleation factor proteoglycan *bgna*, and hedgehog signalling ligand *ihha* that represent some of these clusters identified above. The expression of the gap junction gene *cx43* was also assessed as it regulates fin regeneration growth [41], and within our PPI network, it was connected to the IGF cluster known to modulate bone growth. These amplicons were assessed in regenerating scales from an independent experiment, and all showed similar trends between RNA-seq and qRT-PCR, validating these findings (Fig. 6A). Note, a subset of genes showed similar qRT-PCR profiles between the RNA-seq total RNA and the independent harvest total RNA samples (Additional file 2: Fig. S6). Ontogenetic scales showed weak *col1a1a* expression, predominantly located in the epidermis adjacent to *sp7* positive cells at the posterior edge of the scale (Fig. 6B). In regenerating scales, *col1a1a* promoter activity was elevated along with expanded *sp7* expression covering most of the scale plate. We observed elevated activity at newly forming circuli associated with thickening of the calcified ECM (Figs. 2B and 6B). We performed the same procedure in *col10a1a:Citrine* and *col2a1a:mCherry* double transgenic zebrafish where the *col2a1a* reporter functions as a negative control as it was not a DEG and is associated with cartilage formation. This showed that ontogenetic reporter expression of both transgenes was absent. At 9 dph, only *col10a1a* reporter expression was observed, specifically at the posterior edge of the scale and interestingly also at the newly developing circuli (thicker ECM) located at the anterior region of the scale (Fig. 6C). These findings confirm that the RNA-seq dataset of regenerating scales contains networks with osteo-active factors that actively assemble (nucleate) a calcified collagen-rich matrix.

### Differentially expressed genes are enriched for human orthologues that cause monogenetic skeletal disorders and that associate with polygenetic skeletal traits

**Fig. 7:**
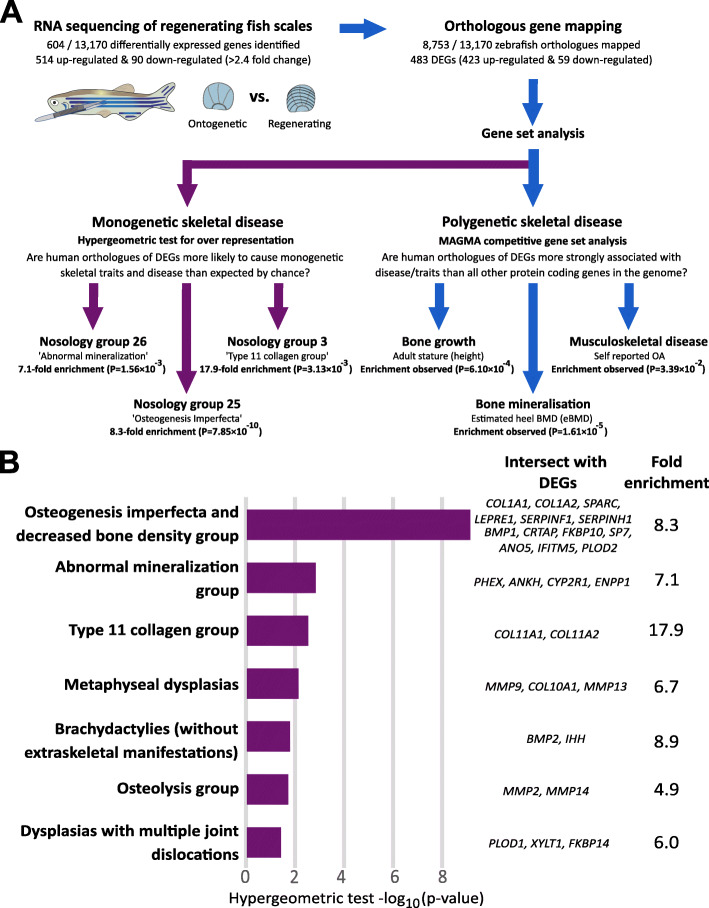
Genes that are upregulated during zebrafish scale regeneration are enriched for genes involved in human musculoskeletal disease. **A** Schematic describing the experimental design used to show that zebrafish DEGs are enriched for genes that cause rare monogenetic skeletal disorders in humans, and for genes that are robustly associated with polygenetic human traits: bone mineral density and height. **B** Bar plot summarising results of enrichment analysis involving genes that that cause different monogenetic dysplasia subgroups. The size of each bar reflects the strength of evidence of enrichment [i.e. hypergeometric-log_10_ (*p*-value). Only dysplasia subgroups with nominal evidence of enrichment (*p*-value < 0.05) are shown. Human gene symbols corresponding to upregulated genes that have human orthologues that cause different monogenetic dysplasia subgroups in humans are listed

We hypothesised that DEGs were likely to be enriched for human orthologues involved in skeletal disorders involving abnormal bone formation. Hypergeometric tests involving the ISDS Nosology and Classification of Skeletal Disorders database [42] revealed that human orthologues of DEGs were 2.8 times more likely to cause any monogenic skeletal dysplasia in humans than expected by chance (*P*=8× 10^−11^) (Fig. 7A). Orthologues of 47 DEGs resulted in one or more primary bone dysplasias in humans when mutated (Additional file 7). Subgroup analysis revealed that DEGs were most strongly enriched for human genes involved in disorders characterised by low bone mass (i.e. Osteogenesis Imperfecta, *P*=8.9 × 10^−10^), and abnormal bone mineralisation (*P*=1.6× 10^−3^). Compelling enrichment for ‘collagen type 11’ and ‘metaphyseal dysplasia’ groups was also detected; however, these observations did not meet our conservative *Bonferroni* corrected significance threshold (*P*=2× 10^−3^) (Fig. 7B and Additional file 7).

MAGMA competitive gene set analysis (GSA) was used to investigate the relationship between genetic variation surrounding human orthologues of 482 mappable DEGs and estimated heel bone mineral density (eBMD) measured in 448,010 white European adults (Fig. 7A). Human orthologues of DEGs were more strongly associated with eBMD than all other human protein coding genes in the genome, suggesting that DEGs were enriched for eBMD associated genes (*P*=1.6× 10^−5^, Table 3, Additional file 8). In a sensitivity analysis, we adjusted for potential confounders, including the set of orthologues that could not be mapped between fish and humans, and for the set of orthologues that was not expressed in ontogenetic and/or regenerating scales. Adjustment reduced the magnitude of enrichment by ~ 18%; however, the set of DEGs remained enriched for BMD associated orthologues (*P*=4.4× 10^−4^). Post hoc permutation analysis suggested that enrichment was attributable to most, but not all DEGs. To further investigate this, DEGs were stratified according to whether they were up- or downregulated, and each set was re-analysed. Enrichment for eBMD associated orthologues was stronger for the 423 upregulated DEGs (*P*=1.4× 10^−6^), whereas the 59 downregulated DEGs did not appear to be enriched (P=0.75) (Additional file 2: Fig. S7A). We repeated the analysis using height and OA and showed that DEGs were enriched for height associated genes (*P*=6× 10^−4^, Fig. 7A, Table 3 and Additional file 2: Fig. S7B). However, post hoc analysis revealed that enrichment was attributable to a small subset of DEGs, rather than all DEGs. Stratified analysis suggested that upregulated DEGs were more strongly enriched for height associated orthologues, whereas no enrichment was observed for downregulated DEGs (Additional file 2: Fig. S7B and Table 3). We observed some evidence of enrichment for OA associated genes (*P*=0.02); however, this observation did not meet our conservative *Bonferroni* corrected significance threshold.

**Table 3:**
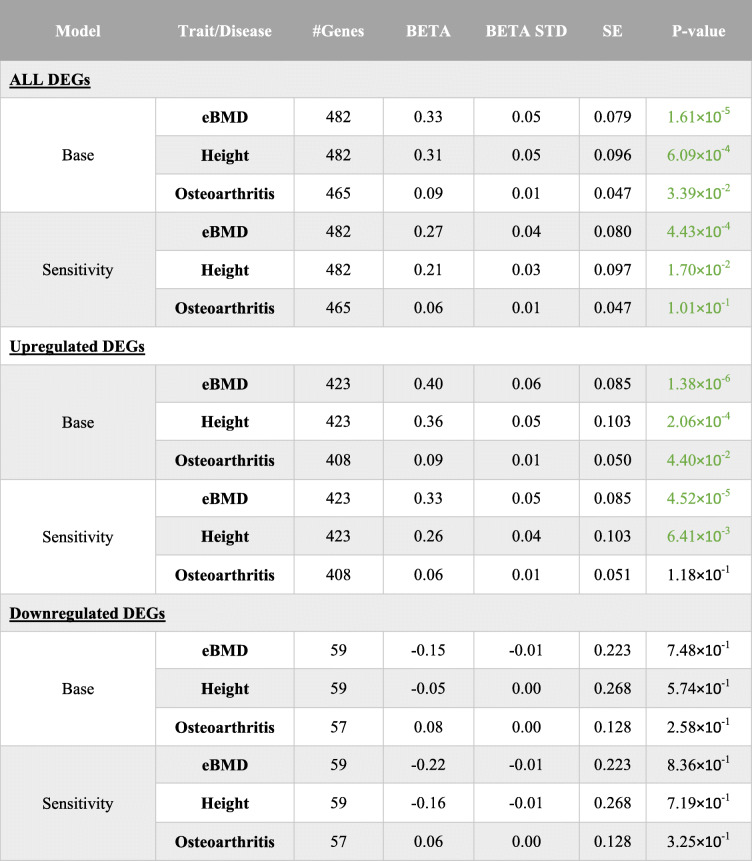
Results of MAGMA competitive gene set analysis involving human polygenetic traits and disease

Model: Statistical models used, with (sensitivity) or without (base) adjustment for the set of genes that could be mapped between zebrafish and humans. Trait: the phenotype or disease trait investigated. #GENES: number of human orthologues of zebrafish DEGs in the gene set. BETA: regression coefficient. BETA_STD: the semi-standardised regression coefficient, corresponding to the predicted change in Z value given a change of one standard deviation in the predictor gene set/gene covariate. SE standard error of BETA. P-value: Strength of evidence to reject the null hypothesis that on average, human orthologs of zebrafish DEGs genes exhibit stronger associations with the corresponding human polygenetic trait / disease than all other protein coding genes in the genome. Green numbers highlight gene sets that have nominal evidence of enrichment (i.e. *p* < 0.05). DEG differentially expressed genes. Full details can be found in the ‘read me’ tab of Additional file 8

### Scale regeneration defects of *spp1* and *col11a2* mutants are consistent with results from human genetic association studies

Our analysis showed that DEGs were enriched for human orthologues that cause monogenetic diseases and are associated with polygenetic traits involving bone growth and mineralisation. We therefore hypothesised that DEGs were also likely to be involved in bone formation during scale regeneration, and endoskeletal development in zebrafish. To investigate this, we selected *spp1* and *col11a2* that were robustly associated in our human genetic studies and validated their predicted role in the zebrafish. We hypothesised that *spp1* was most likely involved in biomineralisation, as *SPP1* was more strongly associated with eBMD (*P*=6× 10^−20^), as compared with height (*P*=5× 10^−3^) or OA (*P*=0.15). In contrast, we hypothesised that *col11a2* was involved in bone growth, size and shape, as *COL11A2* was robustly associated with height (*P*=6× 10^−24^) and OA (*P*=1.9× 10^−6^), but not with eBMD (i.e. *P*=0.02). In fact, the human orthologue of *col11a2* also cause type III Stickler syndrome when mutated, and this disorder is characterised by early-onset OA [43].

**Fig. 8:**
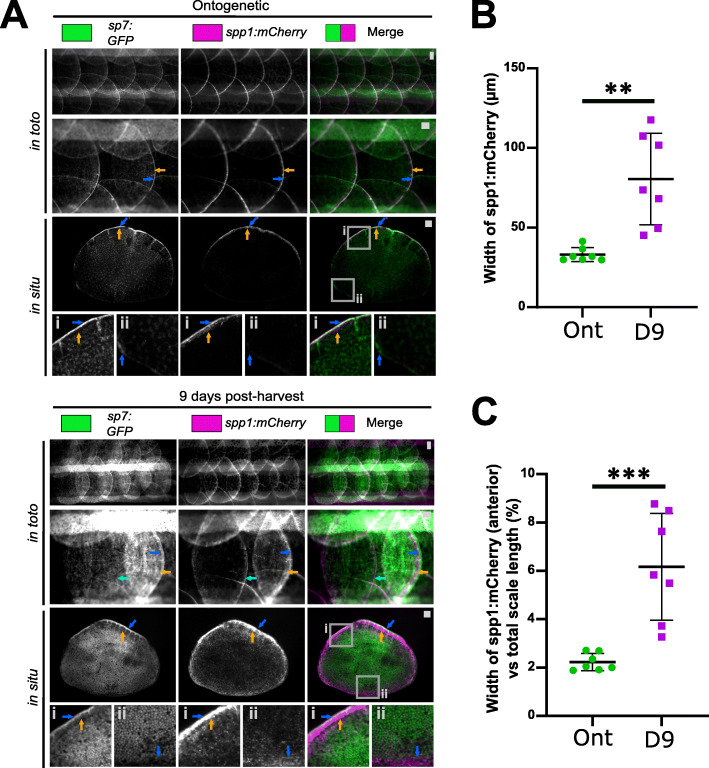
Validation of genes associated with polygenic traits in humans. **A** Images of scales from *in toto* and harvested scales of *sp7:GFP* and *spp1:mCherry* double transgenic fish (ontogenetic: *n* = 5, regenerating: *n* = 4 fish). For overview and inset i, blue arrows indicate high GFP signal and orange the mCherry signal at the posterior edge (inset i) while light blue arrow indicates an ontogenetic scale next to a regenerating scale. Inset ii shows anterior region of the scale with opposite GFP and mCherry signals (blue arrow) in ontogenetic and regenerating conditions. **B** Quantification of width of the mCherry signal at the posterior edge from harvested scales (between blue and orange arrows in panel **C**). **C** Width of mCherry signal normalised by scale length (anterior to posterior). Unpaired two-tailed student t-test p -values are shown on the graph. Scale bar: 100 μm

We first assessed the expression of the two DEGs during scale regeneration. An independent experiment validated that both *spp1* and *col11a2* were upregulated during scale regeneration (Additional file 2: Fig. S8A). We had access to a transgenic reporter of *spp1* (*spp1:mCherry*) and this showed that *spp1* was exclusively localised at the distal rim of the ontogenetic scale, similar to *sp7:GFP-*positive osteoblast localisation marking the leading edge (Fig. 8A). These *sp7-*positive (sub)marginal cells at the rim are classed as more mesenchymal osteoblasts involved in *de novo* bone formation in ontogenetic scales [44]. In regenerating scales, *sp7* expression was seen more broadly but elevated at the posterior edge; followed proximally by an increased *spp1* signal with reduced *sp7* expression in the same area (Fig. 8A). The extent of *spp1* expression was increased compared to ontogenetic scales, implying an enhanced response to bone growth and mineralisation in the regenerating scale (Fig. 8B, C).

**Fig. 9:**
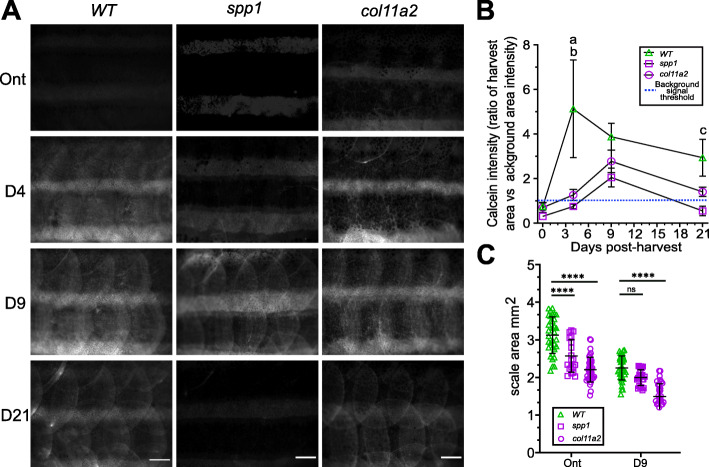
Phenotyping of ontogenetic and regenerating *spp1*^*P160X*^ and *col11a2*^*Y228X*^ homozygous mutant scales. **A** *In toto* images of calcein stained fish showed a delay in scale calcification of predominantly *spp1* mutant fish. **B** A two-way ANOVA analysis on intensity measurements of *in toto* calcein stained fish showed that there was no interaction between genotype and time on calcein uptake (*p*=0.22), but that genotype (*f*(2) = 8.64, *p*< 0.01) and time (*f*(3) = 4.78, *p*< 0.001) independently had a significant effect. Tukey’s multiple comparison showed that *spp1* (a) and *col11a2* (b) mutants had significantly reduced calcein at 4 dph (*p*< 0.01). *spp1* mutants had a reduced tendency at 21 dph (c, *p*=0.09). **C** Scale size was significantly reduced in both mutants (two-way ANOVA, there was no interaction between genotype and time on scale area (*p*=0.10), but that genotype (*f*(1) = 22.49, *p*< 0.0001) and time (*f*(2) = 12.52, *p*< 0.0001) independently had a significant effect). Metrics were derived from Von Kossa stained scales (Additional file 2: Fig. S8). Tukey’s multiple comparison *p*-values are shown on the graph. Scale bar: 100 μm

We stained regenerating scales of *col11a2*^*Y228X*^ and *spp1*^*P160X*^ mutant fish with calcein green. *In toto* images showed that *spp1* mutant regenerating scales bind less calcein compared to wildtype and *col11a2* mutant fish indicative of reduced mineralised matrix formation (Fig. 9A). Quantification of the calcein intensity revealed that both *spp1* and *col11a2* mutants showed a delay and a lower rate of calcein uptake (Fig. 9B). Von Kossa staining on regenerating scales confirmed that *spp1* mutants have mineralisation defects in both ontogenetic and regenerating scales (Additional file 2: Fig. S8B). The *col11a2* mutant scales exhibited uneven mineralisation in the (hypermineralised) epidermal area by displaying areas of hypomineralisation whilst the rim showed increased mineralisation (Additional file 2: Fig. S8B). Both *coll11a2* and *spp1* mutant ontogenetic scales were smaller than wildtype (Fig. 9C). However, while *col11a2* mutants showed a reduced body length and *spp1* mutants were normal length; therefore, when corrected for body size, *spp1* mutants had relatively smaller scales (Additional file 2: Fig. S8C and D). During regeneration, *col11a2* showed growth defects whereas *spp1* mutant scales showed a similar rate of growth at 9 dph (Fig. 9C). These data show that both mutants show exoskeletal phenotypes that are largely consistent with their GSA predictions.

### Endoskeletal phenotypes of *spp1* and *col11a2* mutants are largely consistent with results from human genetic association studies

**Fig. 10:**
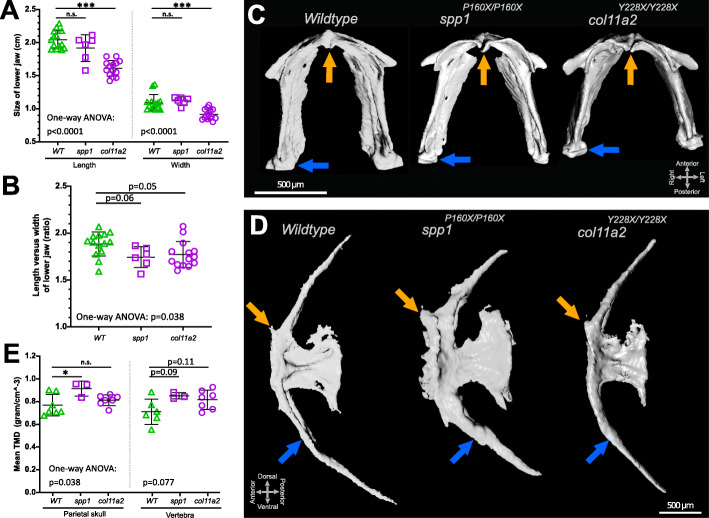
Histomorphology and tissue mineral density measurements on 3D micro-CT images of *spp1*^*P160X*^ and *col11a2*^*Y228X*^ homozygous mutants revealed altered bone structures. **A** Lower jaw size was reduced in *col11a2* mutants (one-way ANOVA: length: (*f*(2) = 33.38, *p*< 0.0001), width: (*f*(2) = 15.06, *p*< 0.0001)). **B** A one-way ANOVA (*f*(2) = 3.636, *p*< 0.05) analysis indicated that lower jaw element proportions showed an altered tendency in both mutants. **C** Ventral view of the segmented lower jaw images. Orange arrow indicates anterior mandibular arch joint and blue arrow shows mandibular arch—palatoquadrate (not visible) joint. **D** Lateral view of segmented images of the first caudal vertebra showing the anterior facet (orange arrow) and haemal arch (blue arrow). **E** Mean tissue mineral density (TMD) calculations and one-way ANOVA analysis of dermal parietal skull (*f*(2) = 4.144, *p*< 0.05) and notochord sheath derived vertebral (*p*=0.077) bone. Tukey’s multiple comparison *p*-values are shown on the graphs

As the exoskeletal collagen template growth process in the form of scales was one of the first biomineralisation processes in evolution to occur, we hypothesised to translate the contrasting patterns of association and scale regenerating defects to endoskeletal sites. Previous adult murine mutants of *Spp1* and *Col11a2* have shown complex (and sometimes conflicting) endoskeletal mineralisation defects and cartilage OA phenotypes respectively [45–48]. However, the adult zebrafish *spp1* and *col11a2* mutant endoskeletal phenotypes have not been described. To consolidate evolutionary conservation of both between aquatic and terrestrial species and between exoskeletal and endoskeletal growth processes we chose to analyse 3D micro-CT renders of both zebrafish mutants. As a proxy for skeletal growth, we evaluated the length of the fish from these micro-CT renders which showed that *col11a2* but not *spp1* mutants were shorter than wildtype fish (Additional file 2: Fig. S9A). Next, we segmented the lower jaw (mandibular arch) and caudal vertebrae of these 3D micro-CT renders and measured several histomorphological parameters using element landmarks as set out in Additional file 2: Fig. S9B-C. These measurements of the lower jaw demonstrated that *col11a2* fish have reduced width and length in the lower jaw whereas *spp1* fish did not show significant changes (Fig. 10A). When we calculated the ratio between length and width, both mutant lines showed a mild alteration in lower jaw element proportion (Fig. 10B). Interestingly, in contrast to wildtype and *spp1* mutants, the *col11a2* mutants exhibited altered joint shape consistent with the predicted OA phenotype in adulthood and as seen in larval jaws previously (Fig. 10C) [49].

We observed a physical thickening of the vertebral arches and anterior shaft of the vertebral centrum in *spp1* mutants (Fig. 10D). However, histomorphological measurements of the centrum, neural arch, and haemal arch of the vertebra did not show significant changes in either mutant line (Additional file 2: Fig. S9D-K). To test whether *spp1* mutants have a mineralisation phenotype, we quantified mean volumetric estimated tissue mineral density TMD (a proxy for BMD) from sites formed via different modes of ossification: the parietal plate of the cranium and the first caudal vertebra as these are formed by intramembranous ossification and by ossification directed by the notochord sheath, respectively. We observed an increase in TMD for *spp1* mutants in the skull, and an increased tendency in vertebrae TMD was seen for both *col11a2* and *spp1* mutants (Fig. 10E). Taken together, our data suggest that genes that are differentially expressed during scale regeneration play a role in wider regulation of skeletal homeostasis at other skeletal sites in the zebrafish, and that mutants in these genes have phenotypes that show consistency with the results from human genetic association studies.

## Discussion

In this study we define, for the first time, the transcriptome of ontogenetic and regenerating zebrafish scales and show that DEGs are enriched for biological pathways involved in osteoblast-mediated bone formation, many of which are conserved in humans. Although fish scales and endoskeletal bone are evolutionary distantly related, based on our findings we conclude that their tissue morphogenesis shares a similar expression profile harbouring osteoanabolic pathways. These matrix-building and calcifying cells (in the literature also referred to as scleroblasts, elasmoblasts, or ameloblasts) that decorate the scale therefore resemble types of osteoblasts [15]. Indeed, recent publications have acknowledged these cells as osteoblast or osteoblast-like cells [13, 19, 44]. By integrating the transcriptomic data with large-scale human genetic association studies, we found that DEGs were enriched for human orthologues that cause monogenetic skeletal diseases, and that associate with polygenetic skeletal disease traits. Our findings suggest that zebrafish scale regeneration has the potential to help us better understand biological function and pathways relevant to bone growth that is often dysregulated in human skeletal diseases.

### Regenerating scales have an expression profile enriched in genes encoding matrix growth and mineralisation

Transcriptomic profiling revealed that regenerating scales upregulate gene expression of many osteoblast genes. The high expression of genes involved in ECM deposition, principally genes regulating collagen synthesis, processing, and deposition fits with the regeneration of the collagen-rich matrix of the scale. We demonstrated that while *col1a1a* is expressed throughout the regenerating scale it is largely excluded from the leading edge labelled by *sp7.* By contrast, *col10a1a* is strongly expressed in scale regions associated with thickening of the calcified matrix in accordance with the role of type X collagen as an early marker of ossifying tissues in the zebrafish [50–52]. Amongst DEG collagens, *col11a2* was one of the highest DEGs in this profile. Type XI collagen is more frequently associated with cartilage matrix formation; interacting with type II collagen to regulate spacing and nucleation of fibrils in the ECM [53, 54]. As well as the collagens themselves, matrix processing genes coding for MMPs were also upregulated. MMPs breakdown the dense type I collagen matrix, which is crucial for tissue homeostasis, release of signalling molecules, and cell migration [55]. We have previously shown that *mmp9* is upregulated during scale regeneration and *mmp9* expression was observed adjacent to newly deposited matrix and TRAP-positive cells [26].

Moreover, we observed transcriptional upregulation of ECM proteoglycans that bind collagens, such as biglycan *(bgna/b*), which are important in regulating collagen nucleation and remodelling [56]. Biglycan has been shown to modulate ECM accessibility, and in regulating bone strength in mice [57, 58]. In addition to collagen related factors, cell adhesion genes, in particular integrin signalling, were enriched and connected to the collagen and MMP networks. Cell adhesion and the ECM are interlinked and both must be tightly regulated during rapid growth [40]. Cell adhesions are heavily modulated to allow cell rearrangements and tissue mechanics in regenerative tissue expansion contexts. For example, in a mammalian epidermal wound healing response, it is crucial for epithelial regeneration [59].

Pathway analysis revealed signalling pathways related to ECM formation and expansion, such as integrin, HH and IGF. The absence of enrichment (note these were also not under-represented) of Wnt, Bmp, and Fgf pathway genes is likely to be associated with the timing of the transcriptomic profile (9 dph). For example, it has been shown that Wnt and Fgf signalling are required for the initiation of scale regeneration, with inhibiting Fgf or canonical Wnt signalling leading to a failure to form scales [19]. HH signalling controls morphogenesis of the scale over a prolonged time period, where the HH ligand secreted from the epidermis regulates the rate of ECM deposition by scale osteoblasts [19, 44].

Our study shows that scales expressed *scpp* repertoire genes that are involved in dentin and endoskeletal bone formation (*spp1* and *sparc*), while *scpp* repertoire and tooth regeneration genes that are associated with enamel matrix hypermineralisation and tooth formation were barely or not differentially expressed [37]. We identified three upregulated teleost-specific *scpp* repertoire genes, *scpp1*, *scpp5* and *scpp7.* Previous reports indicated that *scpp1/scpp5* expression is not restricted to exoskeletal elements; expression was also seen the in jaw and dental tissues, with *scpp1* also expressed in osteoblasts and osteocytes [60]. Whilst the *spp1* genomic region lacks terrestrial *SCPP* ‘bone/dentin’ repertoire *MEPE*, *DSPP*, *SMP1*, and *IBSP* orthologues, we showed that *spp1*, *sparc* and teleost specific *scpp1*, *scpp5* and *scpp7* peaked their temporal expression around 9 dph, coinciding with *col1a1a* transcriptional peaking. This indicates that there is a conserved regulation of expression of SCPP repertoire genes during bone growth and that scales lowly express enamel-related genes.

### Osteoblastic evolutionary conserved gene sets involved in human endoskeletal bone formation are expressed during scale regeneration

The pathways identified are largely conserved in humans, prompting us to investigate whether DEGs were enriched for human orthologues that cause human monogenic skeletal disorders, and that associated with related polygenetic skeletal disease endophenotypes. Hypergeometric tests involving human monogenetic skeletal disorders complemented findings from our gene ontology analysis, suggesting that DEGs were most strongly enriched for human orthologues that skeletal disorders that were broadly characterised by abnormal or decreased bone mineralisation. Suggestive evidence of enrichment for orthologues involved in abnormal cartilage formation and bone growth was also observed. Further analysis involving corresponding human polygenetic skeletal disease traits provided additional evidence that upregulated (but not downregulated) DEGs, were highly enriched for human orthologues associated with eBMD as captured by heel bone ultrasound. Enrichment for height associated genes was also observed for upregulated DEGS; however, post hoc permutation analysis revealed that enrichment was not attributable to all upregulated DEGs, but rather a nested subset of genes that may correspond to one or more biological pathway(s) involved in human bone growth. One possible explanation for the heterogeneity is that DEGs were identified using bulk RNA-sequencing, a method that captures a heterogenous mixture of transcriptome signature profiles that define different cell types, states, and their biological pathways, some of which may or may not be involved in bone growth during scale regeneration. Based on the collective analyses described above, we show that the zebrafish scale harbours *bona fide* osteoblast cell populations. Future studies may therefore benefit by focusing on transcriptomic profiling of separate cell populations from scales at different stages of regeneration to dissect the different pathways responsible for osteo-anabolic growth.

Detailed skeletal phenotyping on zebrafish mutants revealed that *spp1* fish had increased BMD, whereas *col11a2* mutants and developed a lower jaw phenotype that was consistent with OA. These observations were largely consistent with our genetic association analysis that showed that *SPP1* was robustly associated with eBMD*,* but not height or OA, and *COL11A2* was robustly associated with height and OA, but not with eBMD*.* We do however note that *col11a2* mutants had elevated vertebral TMD which was not consistent with results from out genetic association studies. Notably, in humans, mutations in *COL11A2* lead to Stickler syndrome, a condition associated with craniofacial dysplasias, and joint abnormalities that lead to premature OA [43] whilst in mutant mice the cartilage degradation phenotype appears to be milder [45]. Note that COL11A2 heterotrimer partner *COL11A1* is associated with both eBMD and OA traits [61, 62] and causes Stickler syndrome with fragility fractures [63]. The potentially type XI chain α1 and α2 heterotrimer complex disruption could provide an explanation for the uneven mineralisation of the scales and elevated tendency of endoskeletal BMD phenotypes in the *col11a2* mutants. A recent study identified the *col11a2* promotor to show enhanced activity in the regenerating caudal fin bony segments, implying a more global role in (dermal) bone growth besides its known function in cartilage [64].

No clear human phenotype for *SPP1* has been defined yet, but as a *SCPP* repertoire gene, it plays a complex intertwined role in regulating post-inflammatory tissue repair and regeneration of multiple organ systems in vertebrates. We show that *spp1:mCherry* was up-regulated in the regenerating scale adjacent to pre-osteoblastic sp7+ cells which is consistent with previous reports that expression of *spp1* is up-regulated during bone remodelling in both zebrafish fins and mammals [65–67]. Moreover, *spp1* mutants have elevated TMD in the cranial and axial endoskeleton, in line with murine studies where loss-of *Spp1* leads to enhanced mineral content in some parts of trabecular bone [47] and increased cortical thickness [68]. Mouse studies have shown that *Spp1* determines mineralisation rate in the skeleton through regulating *Phospho1* expression [46, 69]. For example, Osteopontin expression is detected during cranial suture closure in both mice and zebrafish, suggesting a role in appropriate timing of pre-osteoblast differentiation as it is often expressed in similar regions positive for *sp7*, *col1a1a1* and *col10a1a* [66, 70, 71]. Outside of its function in skeletal tissue, mammalian *Spp1* is functionally diverse, as it promotes angiogenesis and its expression is also triggered during cutaneous wound healing to control the rate of repair [72, 73]. As Osteopontin is a component of the ECM and possesses integrin binding domains, it can bind various integrins to modulate cell adhesion to the collagen matrix important during i.e. tissue growth [74]. As we see high *spp1:mCherry* expression adjacent to *sp7*+ pre-osteoblasts in the regenerating scale, it implies that *spp1* could play a role in differentiation of these pre-osteoblasts and therefore timing of scale mineralisation and the association with human eBMD is suggestive of a conserved function in control of BMD across species. Indeed, spp1 mutants have mineralisation defects in both ontogenetic and regenerating scales. However, endoskeletal TMD appears to be increased, implying that *spp1* plays an additional role in promoting osteoclastic bone resorption in line with its role in regulating the inflammation response in for example wound healing.

### Scales offer an alternative model to discover novel evolutionarily conserved osteoanabolic genes

The discovery of the WNT pathway inhibitor Sclerostin (SOST) as a high bone mass gene demonstrated the translation health sciences value of understanding the genetic control of bone growth and osteoblast differentiation processes [75]. The combination of human genetic linkage studies subsequent successful modelling in mice and cells showing a transient increase of *Sost* expression in osteoblasts differentiating into osteocytes [75, 76]. This prompted the development of a humanised monoclonal antibody (Romozumab) against SOST which is now an osteo-anabolic osteoporosis therapeutic that reduces fracture risk [77]. Interestingly, previous reports showed that zebrafish scales express osteocyte marker *sost* [24], and we indeed observed expression of *sost*, *wnt3a*, *lrp4*, *lrp5* and *dkk1a* ‘osteocyte markers’ in both ontogenetic and regenerating scale transcriptomes albeit not classed as differentially expressed. Future experiments *in situ* should confirm which cells express these osteocyte markers. While zebrafish scales do not have cells that display cytomorphological characteristics resembling osteocytes, the teleost evolutionary relative bigeye tuna (*Thunnus obesus*) does have thicker trabeculated osteocytic scales that display a lacunocanalicular network [78]. This indicates that it is likely that a subpopulation of the zebrafish scale osteoblasts already express ‘osteocyte markers’ as other previous studies also have shown that there is transcriptional heterogeneity among scale osteoblasts [13, 44].

The common vertebrate ancestors date back more than 400 million years ago allowing morphological and functional divergence between exoskeletal scales and terrestrial endoskeletal bones. In fact, despite this considerable evolutionary distance between teleosts and terrestrial vertebrates (tetrapods), our data not only indicate that the distinct gene expression profiles of scale cells are indeed osteoblast-like profiles, but also demonstrate a striking conservation of osteogenic pathways from teleosts to mammals. When these morphological and functional differences between elasmoid scales and mammalian endoskeletal bones are considered, scales offer an alternative avenue to study a subset of genes involved in bone growth. Since the prevalence of diseases with pronounced bone fragility phenotypes is increasing due to an ageing population there is a need to discover and rapidly test new bone growth candidate genes that could act as drug targets. These multi-factorial diseases have complex genetic and physiological underpinnings that are not well understood. The regenerating zebrafish scale could therefore function as an additional model to study and discover osteo-anabolic factors relevant to human skeletal diseases. The relative abundance of teleost scales (hundreds per fish) and their amenability for imaging make them an ideal model for regenerative studies, and unlike caudal fins, scales can be treated as independent bone units, each harbouring their native complex tissue environment retaining vital intercellular interactions (between osteoblasts and osteoclasts). Scales can be cultured *ex vivo* in a semi-high throughput multi-well format, offering a path to test compounds that could complement established tissue culture and *in vivo* pharmacology studies [32, 79].

## Conclusions

Zebrafish regenerating scales express osteoanabolic gene sets that are evolutionarily conserved and despite the evolutionary distance between exoskeletal and endoskeletal elements, scales show transcriptional similarities with mammalian endoskeletal bone growth. They express human orthologues involved in skeletal disease making the scales an attractive model to identify novel players and their pathways in matrix formation and calcification. Moreover, our analyses provide strong evidence that zebrafish scales are decorated with *bona fide* osteoblast cell populations. We show that integrative analysis involving zebrafish transcriptomics and human genetic association studies is feasible, and that future studies involving zebrafish scales have potential to better our understanding of bone formation in general that could aid studies involving skeletal disease pathogenesis.

## Methods

### Zebrafish husbandry, mutant and transgenic lines

Zebrafish were maintained under normal husbandry conditions [80]. Wildtype AB/TL (University of Bristol, UK) and AB (Radboud University, Nijmegen, NL) strains were used. Mutant lines (AB/TL background, UoB) have been previously described; *col11a2*^*sa18324*^ carries a nonsense mutation causing a premature stop codon at tyrosine position 228 (ENSDART00000151138.3), henceforth called *col11a2*^*Y228X*^ [49] and *spp1*^*CGAT327-330del*^ carrying a deletion leading to a frameshift resulting in a premature stop codon nonsense mutation at proline position 160 (ENSDART00000101261.6), henceforth called *spp1*^*P160X*^ [81]. Transgenic lines were kept in an AB/TL background and full nomenclature as follows: *spp1:mCherry [TgBAC(spp1:mCherry)*^*bsl374*^ [81]], *col2a1a:mCherry* [*Tg(Col2a1aBAC:mCherry)* [50]], *sp7:GFP* [*Tg(Ola.sp7:NLS-GFP)* [82]], *sp7:mCherry* [*Tg(osterix:mCherry-NTRo)*^*pd46*^ [83]], *col1a1a:GFP* [*Tg(col1a1a:EGFP)*^*zf195tg*^ [84]], and *col10a1a:Citrine* [*TgBAC(col10a1a:Citrine)* [85]].

### Elasmoid scale harvesting and imaging

Anaesthetised fish (0.05% (v/v) tricaine methanesulfonate (MS-222) (UoB), 0.1% (v/v) 2-phenoxyethanol (RU)) were put on a wet tissue containing system water and anaesthetic, and scales plucked under a microscope with a watchmaker’s tweezers from the midline of the lateral flanks near the dorsal fin. Fluorescent microscope images of flanks (*in toto*) and single harvested scales (*in situ*) were acquired on a fluorescent stereomicroscope (Leica Microsystems, Germany), using ×2, ×4 and ×8 magnification.

#### In vivo calcein staining

Adult fish were immersed in 40 μM Calcein (Sigma-Aldrich, cat# 154071-48-4) Danieau’s buffer solution (pH 7.4) for two hours and washed in system water for at least 15 minutes prior to imaging. Calcein intensity was measured from *in toto* images of ontogenetic scales and regenerating scales (4, 9. 21 dph). For each image, pixel intensity was measured in ImageJ at locations where two adjacent scales overlap with each other for both non-harvest area (background) and harvest area (4 regions per image). Calcein intensity was calculated using the ratio of harvest area versus background area intensity.

#### Alkaline phosphatase staining

Scales were collected in ALP buffer (100 mM Tris-HCl (pH 9.5), 100 mM NaCl, and 50 mM MgCl_2_) and stained in ALP buffer containing 2% (v/v) NBT/BCIP (Sigma, cat# 11681451001). After a brief wash in deionised water, scales were mounted on a microscope slide containing Mowiol® 4-88 (10% w/v, Sigma-Aldrich cat# 9002-89-5) in glycerol (25% v/v) solution. Images were taken on an upright microscope (Leica).

#### Von Kossa staining and scale morphometric analysis

Plucked scales were washed in deionised water and then incubated in silver nitrate (5% w/v) for 1 h in strong light. After deionised water washes the scales were fixed in sodium thiosulphate (5% w/v) and mounted on a microscope slide containing Mowiol® 4-88 solution.

### RNA isolation, RNA sequencing, transcriptomic mapping and analysis

Approximately 40 scales were collected from a standardised area on the left flank of 1-year-old male zebrafish; the area that extends from just behind the operculum to the implant of the dorsal fin. The area included multiple rows of scales of similar size and shape. Total RNA was isolated from ontogenetic and regenerating scales (*n*=3 fish per group, RU) by using Trizol (Invitrogen) for RNA-sequencing (RNA-seq) and downstream qRT-PCR testing.

RNA-seq was performed by ZF-GENOMICS (Leiden, NL) and involved quality control of total RNA libraries on 2100 expert bioanalyzer (Agilent) that resulted in RIN scores of > 9.2. Illumina RNAseq library preparation involved standard 6 nucleotide adaptor ligation. Paired single read 1 × 50 nucleotide runs (10 million reads; 0.5 Gb per sample) were performed on an Illumina Hiseq2500 system.

#### Transcriptome mapping and differential expression analysis

Raw reads were mapped to the GRCz11 primary assembly (Ensembl version 99) [86] using STAR (version STAR_2.5.4b) software pipeline [87]. The read count table for all genes mapped was obtained from the mapping step and filtered to leave out lowly expressed genes by only keeping genes that had at least 5 mapped reads over all samples. The differential gene expression analysis was performed using the R-package DESeq2 (version 1.28.1) [88] including the medians of ratio normalisation step to account for the bias in sequencing depth/coverage and RNA composition of samples. For determining DEGs, we used a threshold of 1.25 log_2_ fold (2.4-fold) change and a false discovery rate (FDR) of < 0.05. Further details are in Additional file 9.

For downstream analyses, genes were classed as ‘expressed’ in scales when all three ontogenetic and regenerating scale samples produced a > 5 normalised read count (background gene list). Arbitrary threshold for differential expression was set at ±1.25 log_2_ fold and an adjusted *p* value (padj) of ≤0.05 (DEG list).

### Gene ontology enrichment and STRING network analysis

DEG and background expression gene lists’ gene symbols were uploaded to GOrilla [89] using *Danio rerio* and ‘two unranked lists of genes’ as settings. The hierarchical images and Microsoft Excel files were used for figure making. Ensembl IDs of DEGs were analysed with an ‘Overrepresentation Test’ (Released 20200407) using Fisher’s exact test and False Discovery Rate correction in PANTHER Gene Ontology (release 2020-06-01), and PANTHER ‘Pathways’ and ‘Reactome pathways’ (PANTHER version 15.0) software [90].

For STRING (v11) gene network analysis [91], the DEG set (zebrafish gene symbols) was uploaded and interaction score (high, ≥0.7 or medium ≥0.4), number of interactions (one shell with max. 10 interactions), and active interaction sources (all were on) were set. More details and other downstream procedures [92–96] are in Additional file 9.

### Gene set enrichment analysis involving monogenic and polygenic skeletal traits and disease

We used hypergeometric tests in conjunction with ISDS Nosology and Classification of Skeletal Disorders database [42] to identify whether DEGs were enriched for human orthologues of skeletal disease-causing genes. We repeated the analysis focussing on 42 broad skeletal disease classes that had been characterised by the nosology based on having similar clinical, radiographic, and molecular phenotypes. Only 24 of the 42 disorder groups were considered as no DEGs were present in the remaining disorder groups. A Bonferroni corrected significance threshold of *p*< 2× 10^−3^, was used to declare statistical significance (total of 25 tests, assuming a type-1 error rate of *α* = 0.05). MAGMA competitive gene set analysis (GSA) was used to investigate whether DEGs were enriched for orthologues of human genes that were associated with eBMD, height and any form of self-reported or hospital defined OA. GSA encompassed three stages. (Stage 1) Orthologous gene mapping: Zebrafish – human orthologues of all protein coding genes were mapped using dbOrtho (BioDBnet) ortholog converter (https://biodbnet-abcc.ncifcrf.gov/db/dbOrtho.php) using Danio rerio (GRCZ11) as input (‘Gene Symbol’ or ‘Ensembl ID’) and Homo sapiens as output (‘Ensembl ID’) [94]. Only Ensembl IDs generating 1:1 results were kept, and duplicates of zebrafish paralogs (2:1 conversion) were removed leading to a list of single human Ensembl IDs (see Additional file 9). (Stage 2) Gene-based association testing: The association between human protein coding genes and eBMD, height and OA was evaluated separately using the weighted average of two different gene-based tests implemented in MAGMA v1.08 [97]. A Bonferroni corrected gene-wide significance threshold of *p*< 2.5× 10^−6^ was used (19,856 tests, *α* = 0.05). MAGMA gene-based tests were conducted on summary results statistics from a GWAS performed inhouse using BOLT-LMM v2.3.4 [98], correcting each trait for age, sex, genotyping array and ancestry (Additional file 2: Fig. S10 and S11) informative principal components 1 - 20 as previously described (61). GWAS involved high quality genome-wide imputed v3 genetic data (~ 12 million SNPs, INFO > 0.9, MAF > 0.05%) measured in 448,010 white Europeans from the UK-Biobank Study that had both eBMD and height measured [99]. Gene-based tests were conducted on OA using GWAS summary results statistics from the arcOGEN Consortium [62]. (Stage 3) MAGMA competitive GSA: The strength of association of human orthologues of DEGs with each trait was compared to the strength of association of all other protein coding genes in the genome. For each trait, GSA was conducted on all DEGs, upregulated DEGs, and downregulated DEGs. Evidence of enrichment was quantified using a one-sided test of statistical significance, together with a *Bonferroni* corrected *p*< 5× 10^−3^ (i.e. 9 tests, *α*=0.05). Sensitivity analysis was performed by further adjusting for the set of human genes that could not be mapped between human and zebrafish and for the set of human orthologues that was not expressed in our zebrafish experiments. Post hoc permutation analysis was performed for analyses that were suggestive of enrichment. Refer to Additional file 9 for an in-depth description of all methods and datasets used [98, 100–104].

### Quantitative real-time PCR

Five hundred nanograms of total RNA was treated with DNase (1 unit) and reverse-transcribed (random hexamer primers) with SuperScript II (Invitrogen 100 units). iQ SYBR Green Supermix (Biorad) containing 350 nM primer and cDNA was used for amplification (primers and PCR conditions in Additional file 9). Relative expression was calculated based on a normalisation index of two reference genes: *eef1a1l1* and *rpl13*. For comparison of qRT-PCR and RNA-seq expression of amplicons, the average of ontogenetic normalised expression (qRT-PCR) or read count (RNA-seq) were taken and every individual value was compared to the average ontogenetic expression (e.g. read count regenerating scales individual 2/average read count ontogenetic). The *p* values presented in the figures were derived from a two-tailed *t* test (qRT-PCR) and *p*-adjusted (padj) from the DESEQ2 analysis (RNA-seq).

### Micro-computed tomography and tissue mineral density calculations

MicroCT was performed as previously described [105], with estimated tissue mineral density (TMD) calculated as previously described [106]. Briefly, *col11a2*^*Y228X/Y228X*^ (*n*=7), *spp1*^*P160X/P160X*^ (*n*=3) mutant and age-matched WT (*n*=7) zebrafish (1-year-old, all AB/TL strain) were fixed, dehydrated to 70% EtOH and scanned at 21 μm voxel size (scan settings 130 kV, 150 μA, 0.5-s exposure, 3141 projections). Images were reconstructed using NRecon software (version 1.7.1.0), with dynamic ranges calibrated against a scan of hydroxyapatite phantoms (0.25 g cm^−3^ and 0.75 g cm^−3^) scanned with identical parameters. For TMD calculations Aviso software (Avison2020.2; Thermo Fisher Scientific) was used to isolate pixel greyscale values for the skull (parietal) and vertebrae (vertebrae 11–13) and calibrated against the greyscale values of known hydroxyapatite density phantoms (0.25 g cm^−3^ and 0.75 g cm^−3^) to estimate mean TMD values and their standard deviations. Histomorphological assessment of segmented jaw and vertebrae (11–13) elements were determined by measuring the width, length, and depth of the elements (N.B. left and right side of jaw elements were considered as independent measurements) in AVIZO between element landmarks as shown in Additional file 2: Fig. S8A and 8B.

### Graphs and statistical testing

All graphs and data of RNA-seq and downstream functional data were analysed in GraphPad Prism (v 8.4.3). One-way ANOVA (wildtype against mutant conditions), two-way ANOVA (when multiple genotypes or amplicons and time points are involved) or unpaired *t* test (comparing ontogenetic against regenerating scale qRT-PCR expression data) statistical testing was performed. For ANOVA, results of the interaction factor (e.g. time × genotype) and/or independent factors were reported in the figure legends are as follows: ‘f(degrees of freedom) = *F* ratio (DFd), *p* -value’ when ‘statistically significant (set at *p*≤0.050)’ or just *p* value alone if not. A post hoc Tukey’s (*α* = 0.05) multiple comparison test determined statistical difference between specific samples. All graphs show mean with standard deviation. Type of tests performed is presented in the figure legends. *P* values in figures as follows: n.s. > 0.05, * ≤0.05, ** ≤0.01, *** ≤0.001. RNA-seq, GO, STRING pathway, and GSEA statistical analyses are described in their relevant sections.

## Supplementary Information

**Additional file 1.** List of protein coding genes consistently expressed in ontogenetic and regenerating scales. Excel file containing all protein coding genes and expression values that were captured during RNA-seq experiment.

**Additional file 2: **Additional Tables (S1) and Figures (S1-S11). PDF file containing the following: **Table S1.** DEGs identified belonging to isolated clusters (1-4) with no identified gene ontology; **Figure S1.** Ontogenetic and regenerating differential expression values are clustered together; **Figure S2.** Two top down regulated genes are on the same genomic contig but are not the same protein; **Figure S3.** Hierarchical clustering of ‘Molecular Function’ enriched gene ontology terms using GOrilla web interface; **Figure S4.** Hierarchical clustering of ‘Biological Process’ enriched gene ontology terms; **Figure S5.** STRING Network analysis showing high protein-protein interaction connectivity of DEGs; **Figure S6.** Quantitative Real-Time PCR analysis of RNA expression of bone markers; **Figure S7.** MAGMA competitive gene set analysis involving human polygenetic traits and disease; **Figure S8.** Von Kossa staining of spp1 and col11a2 mutant ontogenetic and regenerating scales; **Figure S9.** Histomorphological measurements of skeletal elements from wildtype; **Figure S10.** Scatterplots describing pairwise comparisons of ancestry informative UMAP components; **Figure S11.** Scatterplots describing pairwise comparisons of ancestry informative PCA components.

**Additional file 3.** List of differentially expressed genes. Excel file containing list of genes classed as differentially expressed.

**Additional file 4.** Gene Ontology data file of DEG using GOrilla. Excel file with several tabs containing outputs from GOrilla GO analyses.

**Additional file 5.** Data output files of PANTHER Overrepresentation Test of process / function / component GO accession numbers and PANTHER Pathway analysis of DEGs. Excel file with several tabs containing outputs of various PANTHER analyses.

**Additional file 6.** Data file containing the PANTHER reactome of DEGs. Excel file with output from PANTHER reactome with DEGs.

**Additional file 7.** Summary of results of gene-set enrichment analysis involving DEGs and human orthologues that cause different skeletal dysplasia subgroups in humans when mutated. Excel file containing a table with nosology enrichment analysis.

**Additional file 8.** MAGMA gene level analysis results quantifying the strength of association between human orthologues of DEG’s and estimated bone mineral density, height and osteoarthritis.

**Additional file 9.** Supplement to the Methods. PDF file containing extended methods.

## Data Availability

The raw RNA-sequencing FASTQ data files supporting the conclusions of this article are available in the European Nucleotide Archive (ENA) repository under accession number PRJEB39971 [107]. The downstream analysed RNA-sequencing datasets, GSA datasets, and *in vivo* data supporting the conclusions of this article are included in this published article and its additional information files. Summary statistics used for GSA studies are available in the cited studies in the relevant Method sections. Raw data files can be found in the data.bris repository by searching with the DOI number of this article [108].

## Abbreviations

ALP: Alkaline phosphatase
DEG: Differentially expressed gene
Dph: Days post-harvest
eBMD: Estimated bone mineral density
ECM: Extracellular matrix
FDR: False discovery rate
GO: Gene ontology
GSA: Gene set analysis
IGF: Insulin-like growth factor
LRR: Leucine-rich repeat
MMP: Matrix metalloprotease
MS-222: Tricaine methanesulfonate
MSK: Musculoskeletal
NCAM: Neural cell adhesion molecule
NLR: NOD-like receptor
OA: Osteoarthritis
PCA: Principal component analysis
PPI: Protein-protein interactions
qRT-PCR: Quantitative real-time polymerase chain reaction
RNA-seq: RNA-sequencing
SCPP: Secretory calcium-binding phosphoprotein
SOST: Sclerostin
SPP: Secreted phosphoprotein
SPP1: Osteopontin
TMD: Tissue mineral density
TRAP: Tartrate-resistant acid phosphatase
