# Supplementary material for: Regenerating zebrafish scales express a subset of evolutionary conserved genes involved in human skeletal disease
Source: BMC Biol. 2022 Jan 21;20:21. doi: 10.1186/s12915-021-01209-8 (PMC8780716; doi:10.1186/s12915-021-01209-8)
Supplement: Supplementary file 2 — Additional file 2: Additional Tables (S1) and Figures (S1-S11). PDF file containing the following: Table S1. DEGs identified belonging to isolated clusters (1-4) with no identified gene ontology; Figure S1. Ontogenetic and regenerating differential expression values are clustered together; Figure S2. Two top down regulated genes are on the same genomic contig but are not the same protein; Figure S3. Hierarchical clustering of ‘Molecular Function’ enriched gene ontology terms using GOrilla web interface; Figure S4. Hierarchical clustering of ‘Biological Process’ enriched gene ontology terms; Figure S5. STRING Network analysis showing high protein-protein interaction connectivity of DEGs; Figure S6. Quantitative Real-Time PCR analysis of RNA expression of bone markers; Figure S7. MAGMA competitive gene set analysis involving human polygenetic traits and disease; Figure S8. Von Kossa staining of spp1 and col11a2 mutant ontogenetic and regenerating scales; Figure S9. Histomorphological measurements of skeletal elements from wildtype; Figure S10. Scatterplots describing pairwise comparisons of ancestry informative UMAP components; Figure S11. Scatterplots describing pairwise comparisons of ancestry informative PCA components. [file 12915_2021_1209_MOESM2_ESM.pdf]

Supplemental tables and figures

D.J.M. Bergen *et al.*

Table S1: DEGs identified belonging to isolated clusters (1-4) with no identified gene ontology

Cluster #	*Danio rerio* gene symbol	*Danio rerio* Ensembl ID	Log_2 Fold Change	False Discovery Rate	Wikigene description
**1**	*si:ch211-204c21.1* (*LOC553492*)	ENSDARG00000053091	1.564	4.26E-04	B double prime 1, subunit of RNA polymerase III transcription initiation factor IIIB (2342 aa)
**1**	*myo6b*	ENSDARG00000042141	1.382	1.58E-03	myosin VIb
**1**	*sh3gl3b*	ENSDARG00000007302	1.415	2.04E-02	SH3-domain GRB2-like 3b
**1**	*dnm1b*	ENSDARG00000009281	1.672	2.19E-02	dynamin 1b
**2**	*rhoua*	ENSDARG00000019709	1.821	5.73E-11	ras homolog family member Ua
**2**	*bcr*	ENSDARG00000079286	1.663	2.65E-10	BCR activator of RhoGEF and GTPase
**2**	*gdi1*	ENSDARG00000056122	1.381	1.11E-05	GDP dissociation inhibitor 1
**2**	*arhgef9a*	ENSDARG00000061746	1.943	6.51E-05	Cdc42 guanine nucleotide exchange factor (GEF) 9a
**2**	*srgap1b*	ENSDARG00000045789	2.050	3.74E-04	SLIT-ROBO Rho GTPase activating protein 1b
**2**	*arhgef40*	ENSDARG00000078979	1.387	1.36E-03	Rho guanine nucleotide exchange factor (GEF) 40
**3**	*mvda*	ENSDARG00000099336	-1.836	2.51E-05	mevalonate (diphospho) decarboxylase a
**3**	*mvk*	ENSDARG00000004130	-1.920	8.04E-05	mevalonate kinase
**3**	*hmgcs1*	ENSDARG00000103025	-1.831	0.00137	3-hydroxy-3-methylglutaryl-CoA synthase 1 (soluble)
**3**	*tm7sf2*	ENSDARG00000032816	-1.913	0.000689	transmembrane 7 superfamily member 2
**3**	*sigmar1*	ENSDARG00000011418	-1.500	0.00115	sigma non-opioid intracellular receptor 1
**3**	*cyp46a1.3*	ENSDARG00000089177	2.085	0.000546	cytochrome P450, family 46, subfamily A, polypeptide 1, tandem duplicate 3 (zgc:136808)
**3**	*dhcr7*	ENSDARG00000103226	-2.137	0.000183	7-dehydrocholesterol reductase
**3**	*cyp2r1*	ENSDARG00000056587	2.380	1.35E-08	cytochrome P450, family 2, subfamily R, polypeptide 1
**3**	*cyp24a1*	ENSDARG00000103277	-2.182	0.000212	cytochrome P450, family 24, subfamily A, polypeptide 1
**4**	*mybpc2b*	ENSDARG00000021265	6.520	1.04E-18	myosin binding protein C, fast type b
**4**	*tmod4*	ENSDARG00000020890	1.883	3.91E-05	tropomodulin 4 (muscle)
**4**	*tpm4a*	ENSDARG00000023963	1.276	9.81E-05	tropomyosin 4a
**4**	*tpm4b*	ENSDARG00000019128	1.581	1.88E-04	tropomyosin 4b
**4**	*tpm1*	ENSDARG00000087402	1.408	3.56E-04	tropomyosin 1 (alpha)
**4**	*fibina*	ENSDARG00000039949	1.510	1.34E-02	fin bud initiation factor a
**4**	*myl10*	ENSDARG00000062592	3.180	1.71E-02	myosin, light chain 10, regulatory
**4**	*myl6*	ENSDARG00000115496	1.508	1.25E-02	myosin, light chain 6, alkali, smooth muscle and non-muscle
**4**	*mylk3*	ENSDARG00000076348	2.320	2.92E-03	myosin light chain kinase 3
**n.b.**	*hmgcra* (cluster 3) has been omitted as it was not a DEG and was added by STRING as a 'high confidence predicted functional partner'.				

enrichment in the STRING analysis.

Bergen et al _ supplemental figure 1

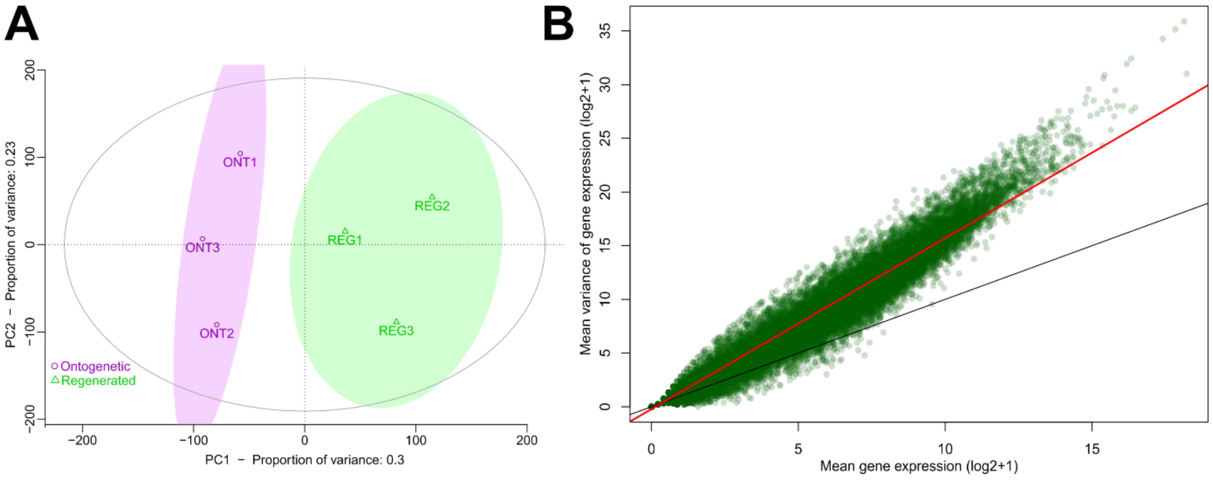

Figure S1: Ontogenetic and regenerating differential expression values are clustered together. A) Principle component analysis (PCA) shows that ontogenetic and regenerating samples show similar variance in their respective groups. **B)** Linear variance analysis showed high level of correlation, Pearson's correlation, r^2=0.9714.

Bergen et al _ supplemental figure 2

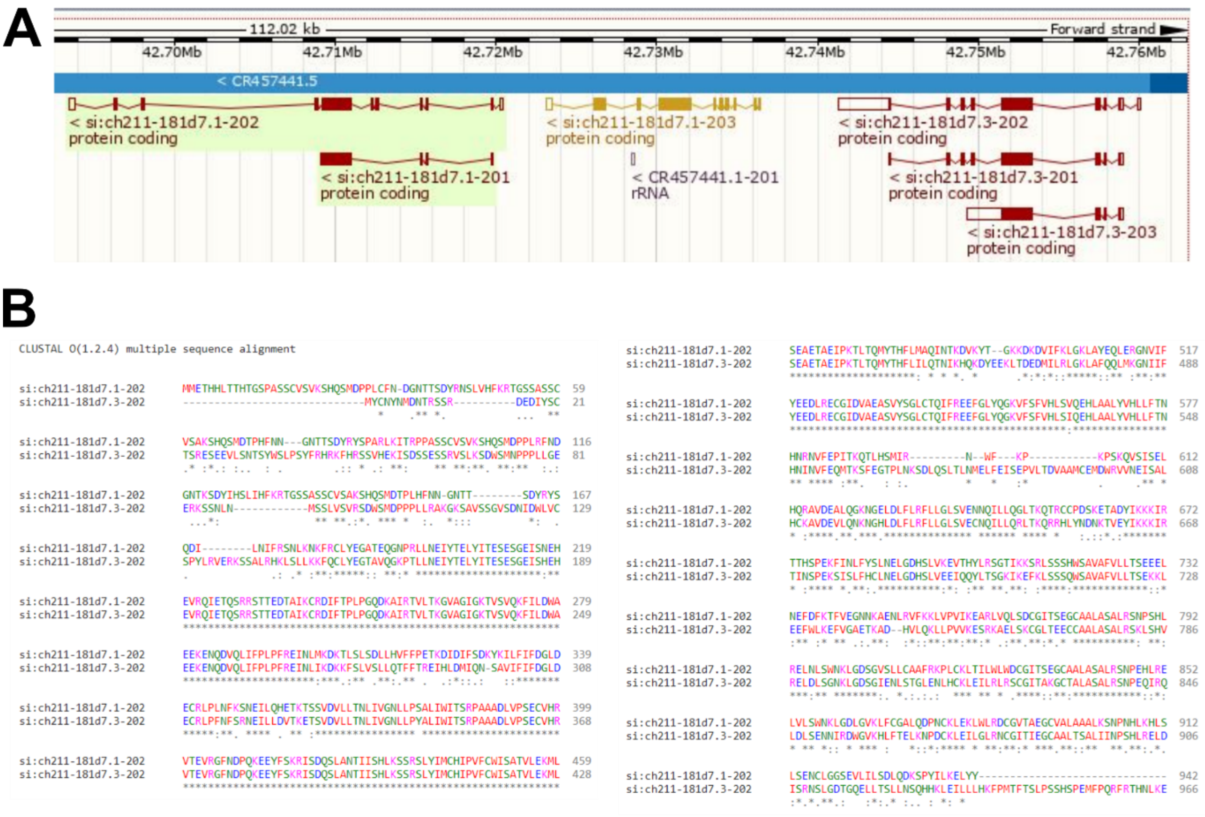

Figure S2: Two top down regulated genes are on the same genomic contig but are not the same protein. A) Screenshot of Ensembl release 100 website showing *si:ch211-181d7.1* and *si:ch211-181d7.3* protein coding genes are on the same genomic contig (CR457441.5) located on chromosome 15. **B)** Clustal Omega protein sequence alignment showing only high sequence similarity ranging from amino acid ~200 to ~580 (*si:ch211-181d7.1*) between the two proteins.

Bergen et al supplemental figure 3

A

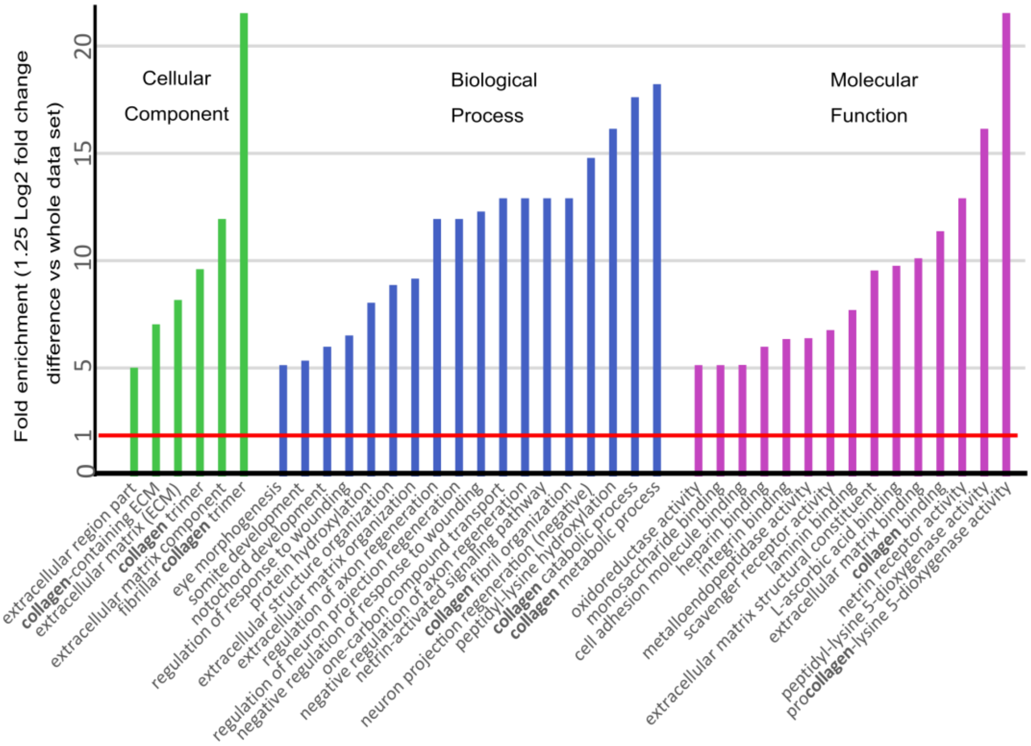

Molecular Function

B

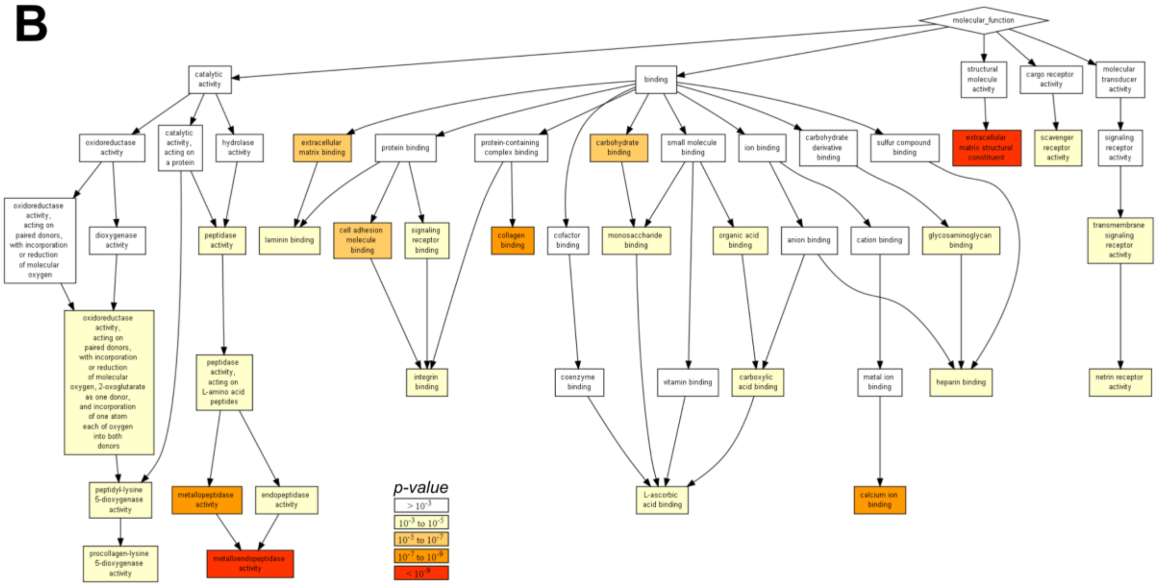

Figure S3: Hierarchical clustering of 'Molecular Function' enriched gene ontology terms using GOrilla web interface. A) Overview of enriched GO terms (>5-fold). Full list can be found in the supplemental data file. **B)** Exported from GOrilla web interface showing GO enrichment of DEGs against background expression dataset.

Bergen et al supplemental figure 4

Biological Process

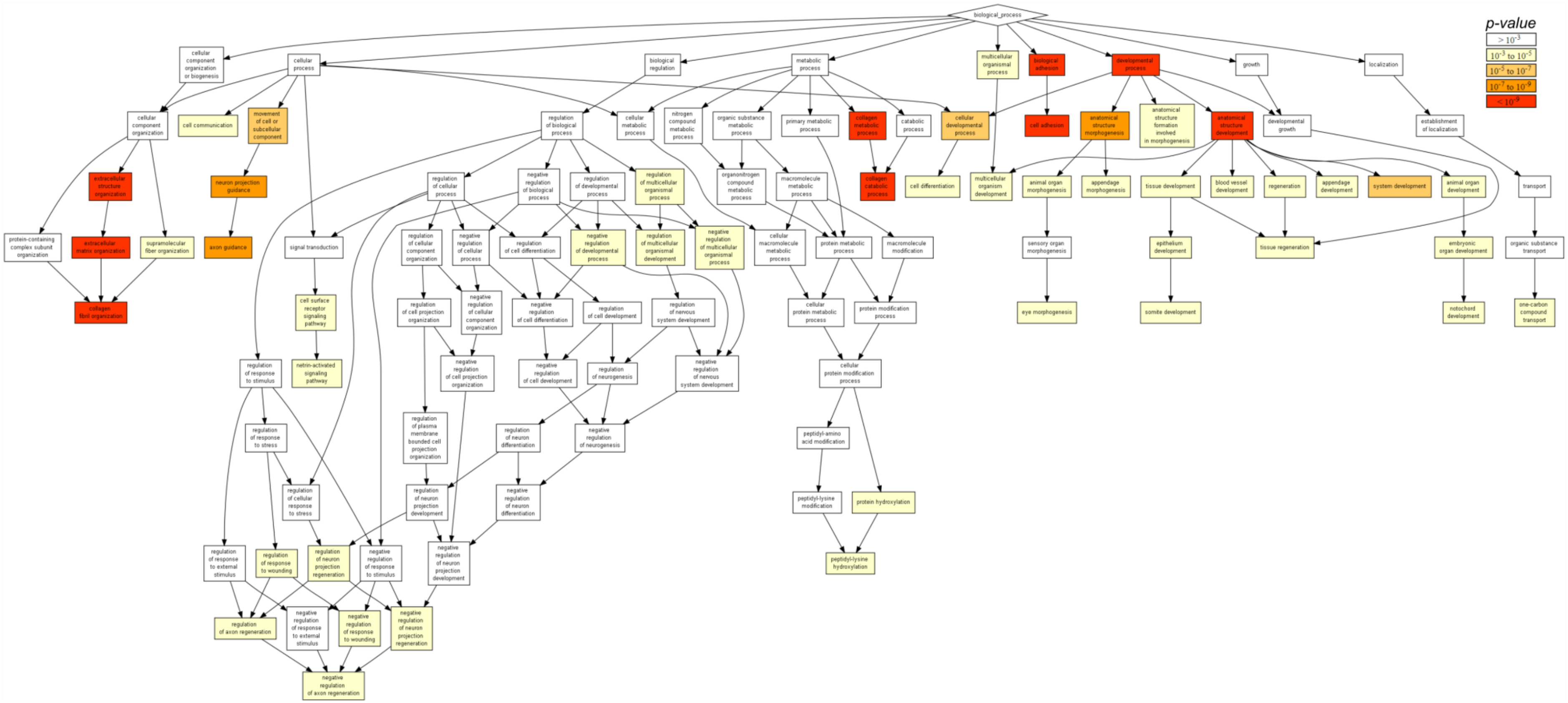

Figure S4: Hierarchical clustering of 'Biological Process' enriched gene ontology terms. Image was exported from the GOrilla web interface showing GO enrichment of DEGs against background expression dataset.

Bergen et al _ Supplemental Figure 5

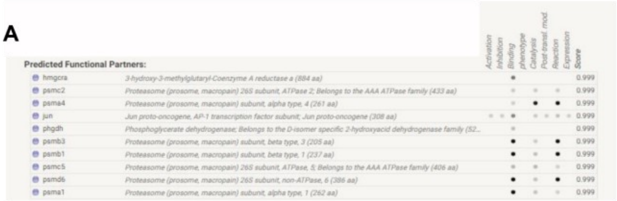

A

Predicted Functional Partners:

Gene	Description	Activation	Inhibition	Binding	phenotype	Catalysis	Post-transl. mod.	Reaction	Expression	Score
hmgcra	3-hydroxy-3-methylglutaryl-Coenzyme A reductase a (884 aa)			●						0.999
psmc2	Proteasome (prosome, macropain) 26S subunit, ATPase 2; Belongs to the AAA ATPase family (433 aa)			●		●		●		0.999
psma4	Proteasome (prosome, macropain) subunit, alpha type, 4 (261 aa)			●		●		●		0.999
jun	Jun proto-oncogene, AP-1 transcription factor subunit; Jun proto-oncogene (308 aa)	●	●	●		●	●	●	●	0.999
phgdh	Phosphoglycerate dehydrogenase; Belongs to the D-isomer specific 2-hydroxyacid dehydrogenase family (52...			●						0.999
psmb3	Proteasome (prosome, macropain) subunit, beta type, 3 (205 aa)			●		●		●		0.999
psmb1	Proteasome (prosome, macropain) subunit, beta type, 1 (237 aa)			●		●		●		0.999
psmc5	Proteasome (prosome, macropain) 26S subunit, ATPase, 5; Belongs to the AAA ATPase family (406 aa)			●		●		●		0.999
psmd6	Proteasome (prosome, macropain) 26S subunit, non-ATPase, 6 (386 aa)			●		●		●		0.999
psma1	Proteasome (prosome, macropain) subunit, alpha type, 1 (262 aa)			●		●		●		0.999

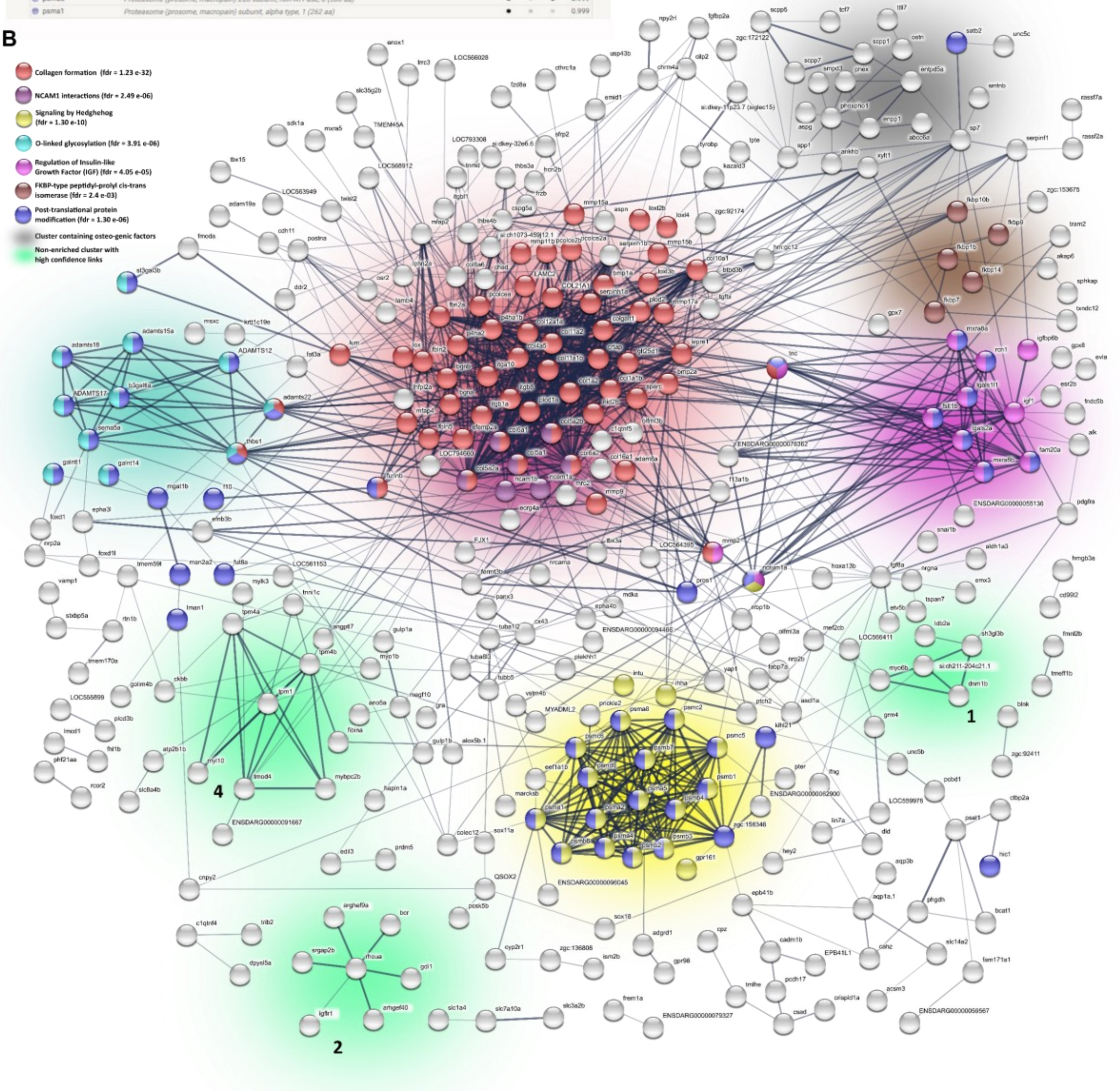

Figure S5: STRING Network analysis showing high protein-protein interaction connectivity of DEGs. A) Proteins added to the network that showed high interaction scores (=0.999) with DEGs that were added to the 'high confidence' (>0.7) and 'medium confidence' (>0.4) protein-protein interaction networks (PPI). **B)** 'Medium confidence' (>0.4) STRING Network of DEGs showing high connectivity between networks. Note that only cluster 2 is not connected to the overall network. PPI enrichment P-value for this network: < 1.0e-16 with a 2.8-fold enrichment in number of connections between nodes.

Bergen et al _ supplemental figure 6

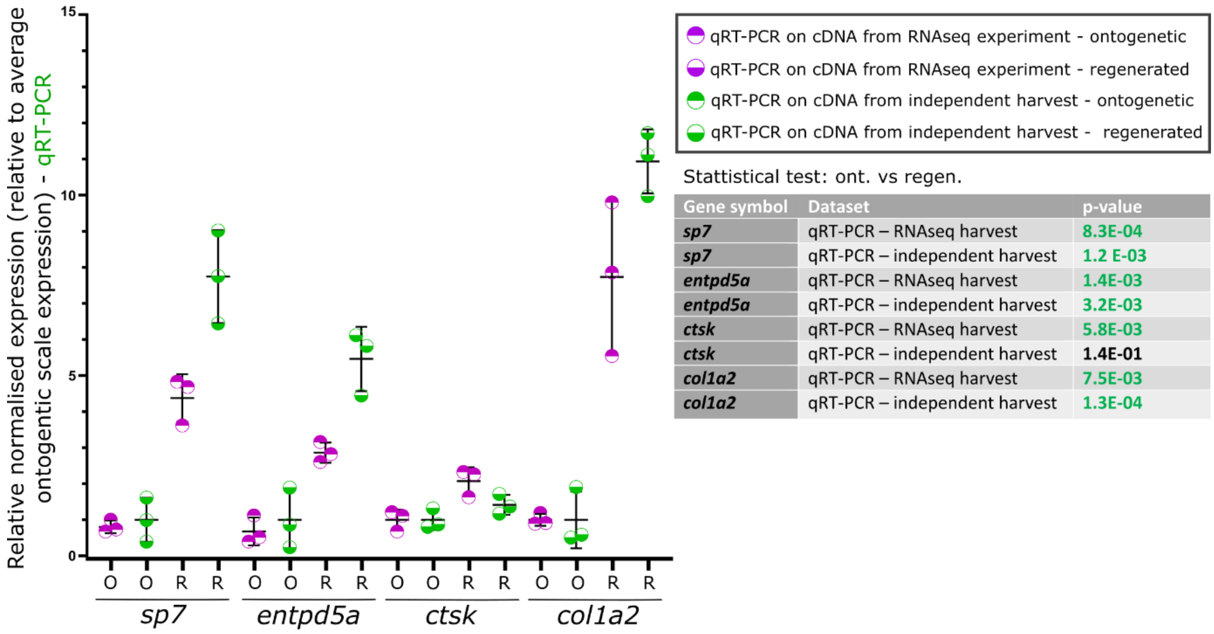

qRT-PCR on cDNA from RNAseq experiment - ontogenetic
qRT-PCR on cDNA from RNAseq experiment - regenerated
qRT-PCR on cDNA from independent harvest - ontogenetic
qRT-PCR on cDNA from independent harvest - regenerated

Stattistical test: ont. vs regen.

Gene symbol	Dataset	p-value
sp7	qRT-PCR – RNAseq harvest	**8.3E-04**
sp7	qRT-PCR – independent harvest	**1.2 E-03**
entpd5a	qRT-PCR – RNAseq harvest	**1.4E-03**
entpd5a	qRT-PCR – independent harvest	**3.2E-03**
ctsk	qRT-PCR – RNAseq harvest	**5.8E-03**
ctsk	qRT-PCR – independent harvest	**1.4E-01**
col1a2	qRT-PCR – RNAseq harvest	**7.5E-03**
col1a2	qRT-PCR – independent harvest	**1.3E-04**

Figure S6: Quantitative Real-Time PCR analysis of RNA expression of bone markers between RNA isolated from the RNA-sequencing scale harvest and an additional independent harvest. Expression profiles show similar expression patterns of the selected amplicons between the two RNA isolations from ontogenetic and regenerating scales.

Bergen et al _ supplemental figure 7

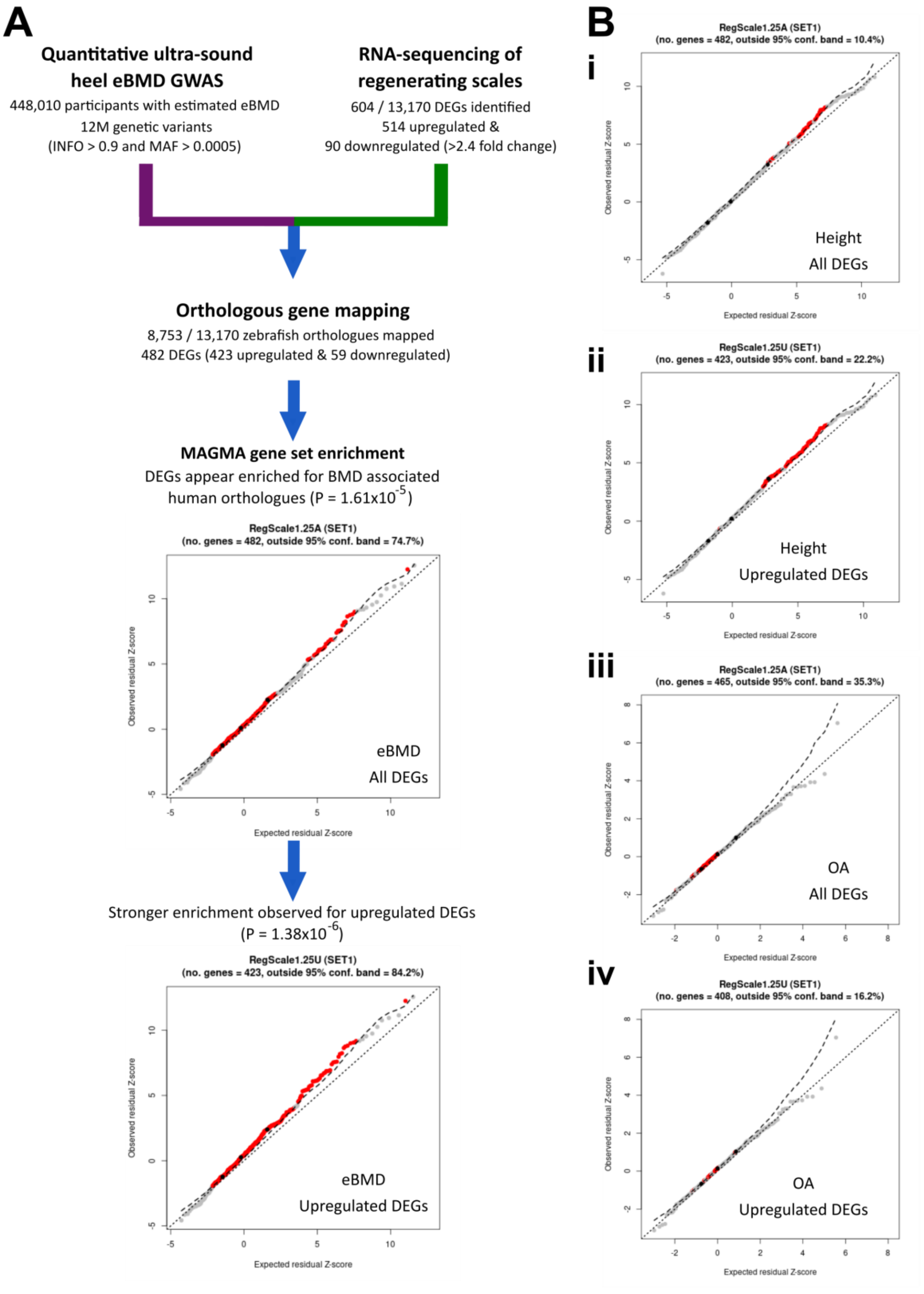

Figure S7: MAGMA competitive gene set enrichment analysis involving human polygenetic traits and disease. (A) MAGMA competitive gene set enrichment analysis was used to investigate the relationship between genetic variation surrounding mappable human orthologs of 482 differentially expressed zebrafish genes and quantitative ultrasound derived heel bone mineral density (eBMD) measured in 448,010 unrelated white European adults from the UK Biobank Study. Strong evidence of enrichment was observed, suggesting that human orthologues of zebrafish DEGs were on

average more strongly associated with the eBMD than all other human protein coding genes in the genome. Post-hoc permutation analysis involved generating quantile-quantile (QQ) plots that contrasted residual Z-scores of differentially expressed orthologue (derived from gene-based tests of association) against its expected Z-score based on the quantile of all orthologues, with the 25th, 50th and 75th percentile denoted by black circles. A one sided upper 95% confidence band was generated to monitor the degree to which the Z-score for each orthologue was likely to deviate from its expected Z-score (denoted by the black dashed line). Orthologues that exceeded this confidence band were coloured in red and all other orthologues were coloured in grey. The proportion of orthologues exceeding the 95% confidence band was reported. Post hoc analysis revealed a mixture of distributions that resulted in a QQ-plot that, (starting from the plot origin) stayed close expectation (denoted by the solid black diagonal line) for most of the differentially expressed orthologues, and deviated upwards towards the end. This pattern, characterised by the late deflection point, suggested that enrichment was not attributable to all differentially expressed orthologues, but rather that the Z-score distribution differed from expectation for a subset of orthologues. To further investigate this finding, orthologues were stratified according to whether they we up- or downregulated, and both sets were re-analysed separately. Stronger enrichment for was observed for the 423 up-regulated orthologues as compared with the set of all differentially expressed orthologues, and no evidence of enrichment was observed for the downregulated orthologues (data not shown). Subsequent post hoc analysis revealed that deviation from expectation occurred earlier and a larger proportion of orthologues exceeded the 95% confidence band (i.e. 40% vs 11% as compared with all differentially expressed orthologues). These findings suggested that human orthologues of many, but not all upregulated DEGs are likely to regulate bone homeostasis in human populations. **(B)** MAGMA gene-set enrichment analysis involving height and self-reported or diagnosed osteoarthritis suggested that DEGs were enriched for human orthologues associated with both traits. Inspection of the QQ plots for height **(i)** and osteoarthritis **(iii)** analysis suggested that enrichment was less pronounced as compared with eBMD, and that in both cases not attributable to all differentially expressed orthologues, To further investigate these findings, orthologues were stratified according to whether they we up- or downregulated, and each set was re-analysed. Enrichment for height associated orthologues was stronger for the 423 up-regulated orthologues as compared with the set of all orthologues, and no evidence of enrichment was observed for the downregulated DEGs (data not shown). Subsequent post hoc permutation analysis revealed a mixture of distributions that was slightly less pronounced **(ii)**, and the deflection point from expectation that started earlier (as compared to the analysis involving all orthologues). These findings suggested that enrichment for height associated orthologues was attributable a moderate proportion (but not all) upregulated orthologues. Analysis involving osteoarthritis revealed that the magnitude of enrichment for upregulated orthologues was similar to the analysis involving all differentially expressed orthologues. QQ plots were similar for both sets of analyses (**ii and iv**), revealing a mixture of distributions suggesting that enrichment was likely attributable to many, but not all differentially expressed orthologues.

Bergen et al Supplemental Figure 8

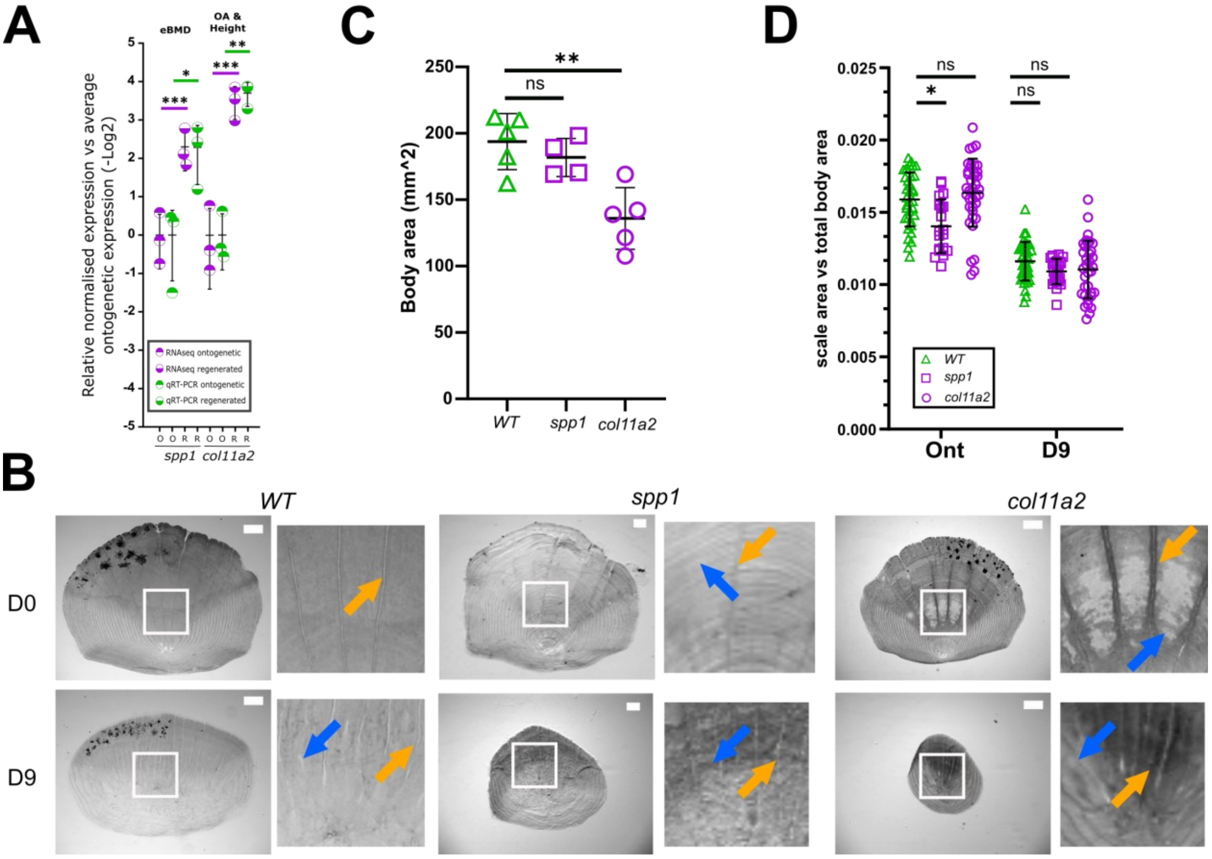

Figure S8: Von Kossa staining of *spp1* and *col11a2* mutant ontogenetic and regenerating scales. **A)** Q-RT-PCR and RNA-seq expression levels (relative to average ontogenetic expression) of *spp1* and *col11a2* DEGs. Statistical threshold of $p<0.05$ (unpaired t-test for qRT-PCR and false discovery rate for RNA-seq). **B)** Upright microscope images of mounted Von Kossa stained scales. Insets are of the central + epidermal (hypermineralised) area of the scale. Orange arrows indicate mineralisation in central + epidermis area groove with more intense silver nitrate labelling in *col11a2* mutants. Blue arrows indicate reduced (*spp1* mutant) or uneven (*col11a2* mutant). **C)** *Col11a2* mutants were significantly smaller. Body surface was measured in FIJI, one-way ANOVA: $f(2) = 11.11$, $p<0.01$ **D)** Each individual scale area was corrected for the corresponding individual's body area (panel B). Ontogenetic *spp1* mutant scales were significantly smaller compared to body surface. Two-way ANOVA showed that there was a significant interaction between time and genotype on scale area corrected for body area: $f(4) = 4.42$, $p<0.01$. Scales (n= 5 to 9 per individual) were harvested from n=5, n=4, and n=5 for wildtype, *spp1,* and *col11a2* genotypes respectively. Scale bar is 100 µm.

Bergen et al Supplemental Figure 9

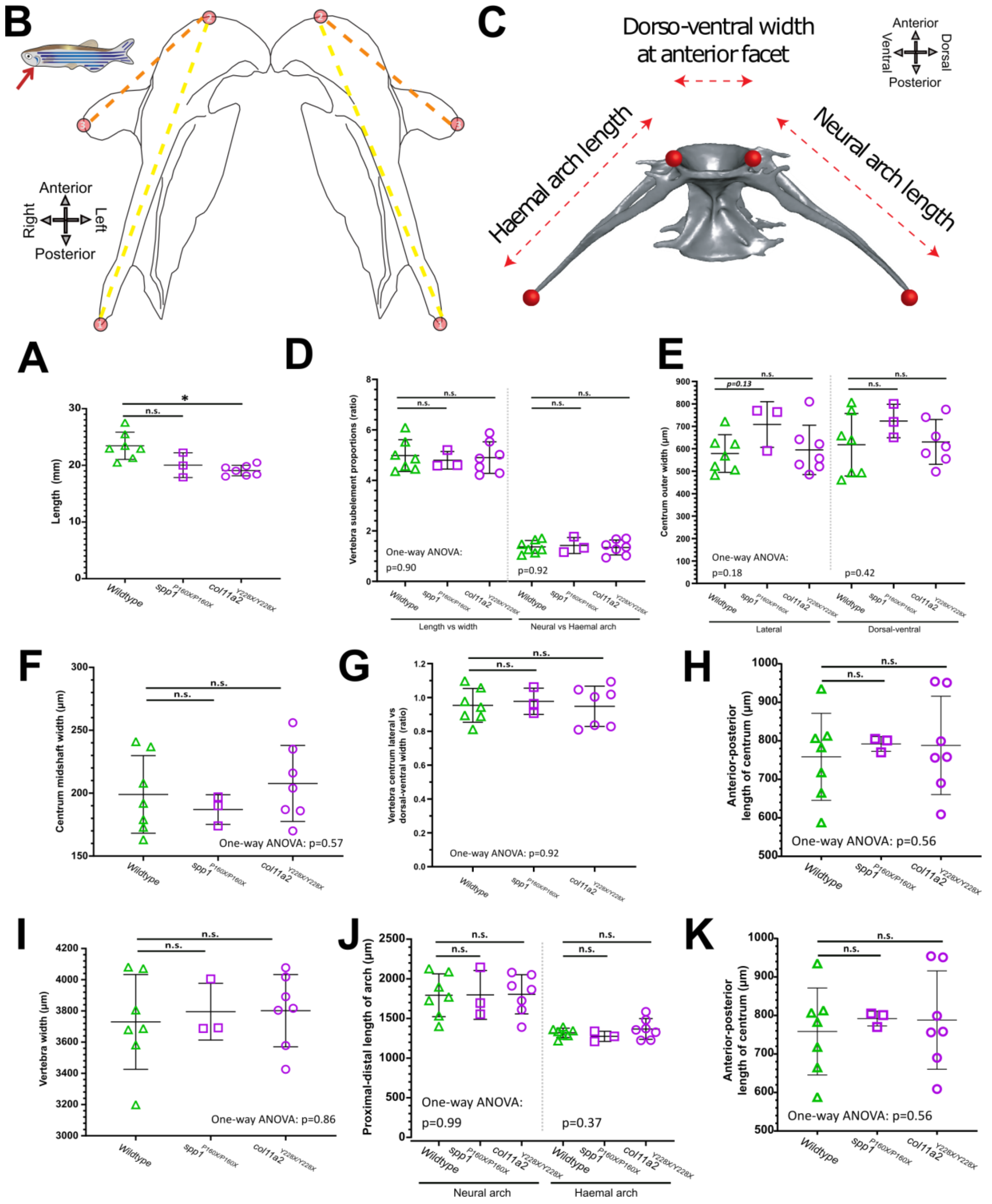

Figure S9: Histomorphological measurements of skeletal elements from wildtype, *col11a2*$^{Y228X/Y228X}$ and *spp1*$^{P160X/P160X}$ fish. A) Mean axial skeleton length taken from CT scans. **B)** Schematic drawing of the landmark points to measure histomorphological parameters of the ventral lower jaw (red arrow). Landmark point 1-2 is length, and 2-3 is width. **C)** Landmark points of a representative 3D micro-CT image from a caudal vertebra used for measuring histomorphological parameters in this study. **D-K)** Measurements of the various caudal vertebrae (number 11-13) element parameters of wildtype, *col11a2*$^{Y228X/Y228X}$ and *spp1*$^{P160X/P160X}$ fish. Mean values of length (anterior to

posterior) and mean width (for whole vertebra element: haemal arch length + neural arch length + anterior facet centrum width) as set out in panel B landmark schematic of each individual were used.

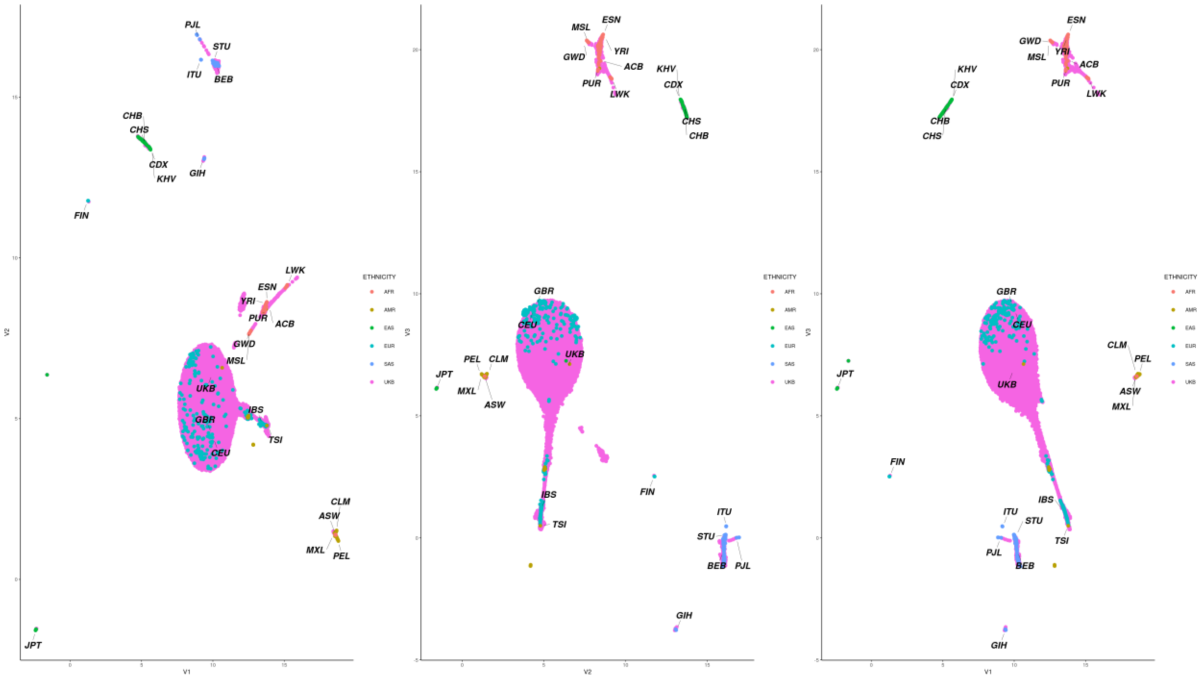

Figure S10: Scatterplots describing pairwise comparisons of ancestry informative UMAP components V1,V2 and V3. Data points represent UK-Biobank Study participants that are coloured in pink. 1000G participants are coloured according to their predefined ethnic group. Samples from the UK-Biobank sample are annotated using "UKB". Other 1000 genomes populations are annotated using the following: CHB=Han Chinese in Beijing, China, JPT=Japanese in Tokyo, Japan, CHS=Southern Han Chinese, CDX=Chinese Dai in Xishuangbanna, China, KHV=Kinh in Ho Chi Minh City, Vietnam, CEU=Utah Residents (CEPH) with Northern and Western European Ancestry,TSI=Toscani in Italia, FIN=Finnish in Finland, GBR=British in England and Scotland, IBS=Iberian Population in Spain, YRI=Yoruba in Ibadan, Nigeria, LWK=Luhya in Webuye, Kenya, GWD=Gambian in Western Divisions in the Gambia, MSL=Mende in Sierra Leone, ESN=Esan in Nigeria, ASW=Americans of African Ancestry in SW USA, ACB=African Caribbeans in Barbados, MXL=Mexican Ancestry from Los Angeles USA, PUR=Puerto Ricans from Puerto Rico, CLM=Colombians from Medellin, Colombia, PEL=Peruvians from Lima, Peru, GIH=Gujarati Indian from Houston, Texas, PJL=Punjabi from Lahore, Pakistan, BEB=Bengali from Bangladesh, STU=Sri Lankan Tamil from the UK, ITU=Indian Telugu from the UK.

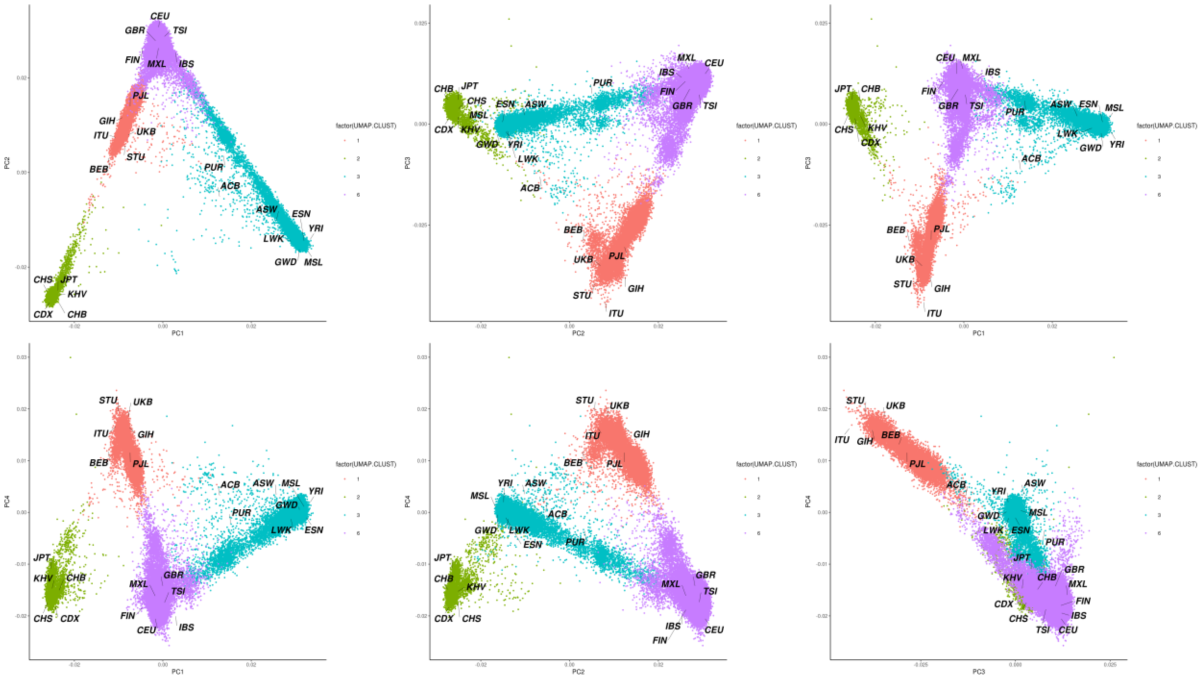

Figure S11: Scatterplots describing pairwise comparisons of ancestry informative PCA components PC1, PC2, PC3 and PC4. Data points represent UK-Biobank Study participants and 1000G participants that are coloured according to their UMAP defined ethnic clusters. Purple – European, Red – American, Green – South Asian and Turquoise – African. Individuals that did not cluster with any 1000G individuals were removed from the analysis (N=2,725). Samples from the UK-Biobank sample are annotated using “UKB”. Other 1000 genomes populations are annotated using the following: CHB=Han Chinese in Beijing, China, JPT=Japanese in Tokyo, Japan, CHS=Southern Han Chinese, CDX=Chinese Dai in Xishuangbanna, China, KHV=Kinh in Ho Chi Minh City, Vietnam, CEU=Utah Residents (CEPH) with Northern and Western European Ancestry,TSI=Toscani in Italia, FIN=Finnish in Finland, GBR=British in England and Scotland, IBS=Iberian Population in Spain, YRI=Yoruba in Ibadan, Nigeria, LWK=Luhya in Webuye, Kenya, GWD=Gambian in Western Divisions in the Gambia, MSL=Mende in Sierra Leone, ESN=Esan in Nigeria, ASW=Americans of African Ancestry in SW USA, ACB=African Caribbeans in Barbados, MXL=Mexican Ancestry from Los Angeles USA, PUR=Puerto Ricans from Puerto Rico, CLM=Colombians from Medellin, Colombia, PEL=Peruvians from Lima, Peru, GIH=Gujarati Indian from Houston, Texas, PJL=Punjabi from Lahore, Pakistan, BEB=Bengali from Bangladesh, STU=Sri Lankan Tamil from the UK, ITU=Indian Telugu from the UK.
