# Supplementary material for: Regenerating zebrafish scales express a subset of evolutionary conserved genes involved in human skeletal disease
Source: BMC Biol. 2022 Jan 21;20:21. doi: 10.1186/s12915-021-01209-8 (PMC8780716; doi:10.1186/s12915-021-01209-8)
Supplement: Supplementary file 9 — Additional file 9. Supplement to the Methods. PDF file containing extended methods. [file 12915_2021_1209_MOESM9_ESM.pdf]

Additional Methods

Dylan J.M. Bergen *et al.*

Transcriptome Mapping

The genomic FASTA sequence, cDNA and GTF annotation files of the latest release (version 99) of reference genome assembly of zebrafish were downloaded from Ensembl (Genome Reference Consortium Zebrafish Build 11). Quality control of the raw reads and adapter trimming was performed by the sequencing vendor. The reference genome was indexed using the genomeGenerate function of STAR (version STAR_2.5.4b) (1) with the following command and parameters:

STAR --runMode genomeGenerate --runThreadN 16 --genomeDir Genome_Ref --genomeFastaFiles GCF_000002035.6_GRCz11_genomic.fna --sjdbGTFfile GCF_000002035.6_GRCz11_genomic.gtf --sjdbOverhang 50

Next, mapping of the paired reads (50 bps) from samples to the reference genome was performed using shell scripting and STAR aligner with the following command and parameters:

for i in {1..2}; do r1="Read1.fastq" r2="Read2.fastq" STAR --genomeDir Genome_Ref --runThreadN 328 --readFilesIn $r1 $r2 --outFileNamePrefix Sample_ONT$i.aligned --outSAMtype BAM SortedByCoordinate --quantMode Sample_ONT1_TranscriptomeSAM; done

The raw read counts were directly obtained from the –quantMode functionality of STAR and genes with zero counts over all samples were removed using R programming language (R version 3.6.3) We obtained a uniform read mapping across all of our samples, with more than 70% of uniquely reads mapped, therefore no further filtering of the data was required.

Differential Expression Analysis

The read count table for all gene mapped was obtained from the mapping step and filtered to leave out lowly expressed genes by only keeping genes that had at least 5 mapped reads over all samples. The differential gene expression analysis was performed using the R-package DESeq2 (version 1.28.1) (2) including the medians of ratio normalisation step to account for the bias in sequencing depth/coverage and RNA composition of samples. Using the built-in function *DESeqDataSetFromMatrix*, the counts data was loaded with the following parameters:

fitType=local, test=Wald, and the batch effect correction included in the design formula.

Linear Variance Analysis

The variance of gene expression across all samples (ontogenetic and regenerative scales) was calculated using the R-package matrixStats (version 0.56.09) and an in-house R script (R version 3.6.3) (see Additional R SCRIPT below).

Principal Component Analysis

The clustering of the samples based on their variance in gene expression was performed using the R-package pcaGoPromoter (version 3.11) and an in-house R script (R version 3.6.3) (see Additional R SCRIPT below).

Volcano plot

Data from the background expression list (**Additional file 1**) in Excel was converted to a tab delimited text file containing -$\log_{10}$ transformed 'padj' values ('minus.log10.padj') and a volcano plot was plotted in R (see Additional R SCRIPT below).

R scripts for Volcano plotting, Linear Variance and Principal Component Analyses

##Linear Variance Analysis R functions

```
library(matrixStats)
variance_regression <- function(as.matrix(Normalized-expression-matrix-from-STAR)){
  dis <- data.frame()
  for (i in 1: nrow(as.matrix(Normalized-expression-matrix-from-STAR)){
    dis[i,1] <- rowMeans(as.matrix(Normalized-expression-matrix-from-STAR)[i, , drop=FALSE])
    dis[i,2] <- matrixStats::rowVars(as.matrix(Normalized-expression-matrix-from-STAR)[i, , drop=FALSE])
  }

  plot(log2(dis[,1]+1), log2(dis[,2]+1),pch=16, xlab="Mean gene expression (log2+1)", ylab="Mean variance of gene expression (log2+1)", main="Variance of gene expression\nacross all samples", col=grDevices::rgb(0,100,0,50,maxColorValue=255))
  abline(0, 1)
  abline(lm(log2(dis[,2]+1)~log2(dis[,1]+1)), lwd=2, col="red")
  print(cor.test(log2(dis[,1]+1), log2(dis[,2]+1)))

  return(dis)
```

```
}

##Principal Component  Analysis and Plot functions
library(pcaGoPromoter)
pca<- function(expmatrix, groups, PCs = c(1,2), main = "PCA plot"){

  #PCA
  pcaOutput <- pcaGoPromoter::pca(expmatrix, printDropped = FALSE, scale=TRUE, center=TRUE)

  #Plot
  pcaGoPromoter::plot.pca(pcaOutput, groups, PCs = PCs, printNames = TRUE, symbolColors = TRUE, plotCI = TRUE, main=main)
}
```

##Volcano plot

```
res <- read.table("Supp-data-file-1_Back-genes-expr_V3.txt", sep = '\t', header=TRUE)
head(res)
library(calibrate)
with(res, plot(log2FoldChange, minus.log10.padj, pch=20, main="Volcano plot", xlim=c(-7,7), ylim=c(0, 20)))
with(subset(res, padj<.05 ), points(log2FoldChange, minus.log10.padj, pch=20, col="blue"))
with(subset(res, abs(log2FoldChange)>1.25), points(log2FoldChange, minus.log10.padj, pch=20, col="magenta"))
with(subset(res, padj<.05 & abs(log2FoldChange)>1.25), points(log2FoldChange, minus.log10.padj, pch=20, col="green"))
with(subset(res, padj<.01 & abs(log2FoldChange)>3), textxy(log2FoldChange, minus.log10.padj, labs=Gene, cex=.5))
with(subset(res, padj<.01 & abs(log2FoldChange)>2 & abs(log2FoldChange)<3), textxy(log2FoldChange, minus.log10.padj, labs=Gene, cex=.5))
```

STRING Network settings

Unconnected STRING network (v11) nodes were excluded, and nodes were colored according to their grouped protein function. Protein function was determined by association with KEGG Pathways (*Danio rerio*, dre04510), Reactome Pathways (*Danio rerio*, DRE-1474290, DRE-3000178, DRE-5358351, DRE-419037, DRE-5173105, DRE-381426, DRE-1592389), or PFAM Protein Domains (PF00090, PF07645) database inputs and these were selected to visualize clusters based on their assigned biological function. Clusters were manually untangled and moved for visual purposes and kept as similar as possible.

Gene contig, protein sequence alignment and literature look-up

ENSDARG00000068621 (*si:ch211-181d7.3*) and ENSDARG00000088274 (*si:ch211-181d7.1*) genes were searched in Ensembl (release 100) and an ortholog search was performed to determine if mammalian orthologues exist. Protein alignment and domain look-up was

performed on Clustal Omega (v1.2.4)(3) and UniProt (v Aug-2020; accession numbers R4GE51 and E7FA88)(4) web interfaces respectively.

Gene set enrichment analysis involving human monogenic and polygenic skeletal traits and disease

Orthologue mapping

Ensembl IDs and gene symbols from differentially expressed (**Additional file 2**), background (**Additional file 1**), and whole genome (Ensembl release 99) genes lists were uploaded to dbOrtho (BioDBnet) ortholog converter (https://biodbnet-abcc.ncifcrf.gov/db/dbOrtho.php) using *Danio rerio* (GRCZ11) as input ('Gene Symbol' or 'Ensembl ID') and *Homo sapiens* as output ('Ensembl ID') (5). Only Ensembl IDs generating 1:1 results were kept, and duplicates of zebrafish paralogs (2:1 conversion) were removed leading to a list of single human Ensembl IDs.

Zebrafish orthologues that were successfully converted to human orthologues were subjected to exclusion criteria that addresses the existing evolutionary genomic complexity between humans and zebrafish due to an additional teleost genome duplication event leaving a subset (~20-25%) of genes with extra paralogs (6, 7). Hence, only zebrafish genes that mapped one-to-one (e.g. *sp7* to *SP7*) or two-to-one (e.g. *ncam1a* and *ncam1b* to *NCAM1*, in this case duplet Ensemble IDs were removed to have a unique human Ensembl ID) to the human genome.

Monogenetic disease

In conjunction with ISDS Nosology and Classification of Skeletal Disorders database (8), evidence of enrichment for human monogenic skeletal disease-causing genes was examined using a Hypergeometric test, using the following parameters: (A) Successes in sample - the number of zebrafish genes that were differentially expressed and that map to unique human orthologues and cause one or more skeletal disorders (either total or within each individual nosology-defined skeletal disorder groups). (B) Sample size - the number of differentially expressed zebrafish genes within the population of genes that were expressed in ontogenetic and/or regenerating genes that map to human orthologues. (C) Successes in population - the number of zebrafish genes that were expressed in ontogenetic and/or regenerating scales and that map to unique human orthologues and cause one or more skeletal disorders (either total or within each individual nosology-defined skeletal disorder groups). (D) Population - the number of zebrafish genes that are expressed in ontogenetic and/or regenerating scales and that map to

human orthologue. Estimates of enrichment were obtained using the formulae provided by the online Hypergeometric p-value calculator (https://systems.crump.ucla.edu/hypergeometric/).

Polygenetic traits and disease

Information about UK Biobank Resource

UK Biobank is a large-scale biomedical database and research resource containing genetic, lifestyle and health information from half a million UK participants (9). UK Biobank's database, which includes blood samples, heart and brain scans and genetic data of the 500,000 volunteer participants, is globally accessible to approved researchers who are undertaking health-related research that's in the public interest. The genotype and phenotype data are available upon application to the UKB (http://www.ukbio-bank.ac.uk/).

UK Biobank recruited 500,000 people aged between 40-69 years in 2006-2010 from across the UK. With their consent, they provided detailed information about their lifestyle, physical measures and had blood, urine and saliva sample collected and stored for future analysis. UK Biobank's research resource is a major contributor in the advancement of modern medicine and treatment, enabling better understanding of the prevention, diagnosis and treatment of a wide range of serious and life-threatening illnesses – including cancer, heart diseases and stroke.

UK Biobank is generously supported by its founding funders the Wellcome Trust and UK Medical Research Council, as well as the Department of Health, Scottish Government, the Northwest Regional Development Agency, British Heart Foundation and Cancer Research UK. The organisation has over 150 dedicated members of staff, based in multiple locations across the UK.

MAGMA and Datasets used for the analysis

MAGMA competitive gene-set analysis (10) was used to investigate whether human orthologues of zebrafish DEGs were more strongly associated with polygenetic traits/disease, than all other protein coding genes in the human genome.

A subset of Europeans individuals from the UK-Biobank study (9) was used for MAGMA GSA involving eBMD and height. Ancestry assignment of UK-Biobank participants was performed as follows: The UK Biobank sample was projected onto the first 20 principal components estimated from the 1000 Genomes Phase 3 (1000G) project (11, 12) (where ancestry was known) using GCTA version 1.93.2 (13). Projections used a curated set of 38,512 LD-pruned HapMap 3 Release 3 (HM3) (12) bi-allelic SNPs that were shared between the 1000G and UK

Biobank genotyped datasets (i.e. MAF > 1%, minor allele count > 5, genotyping call rate > 95%, Hardy-Weinberg P > $1x10^{-6}$, and regions of extensive LD removed). Uniform Manifold Approximation and Projection for Dimension Reduction (UMAP) was used in conjunction with the first 20 principal components to cluster 486,445 individuals using the following parameters: min_dist=0.0001, n_components=3, n_neighbors=45, random_state=10293082. UK-Biobank participants that clustered together with the 1000G European sub-populations were manually identified by visual inspection (N=461,920) and used for downstream genetic analyses (**Additional File 2, Fig. S10** and **S11**).

Gene-based tests of association

MAGMA gene-based tests were conducted on summary results statistics from a GWAS performed inhouse using BOLT-LMM v2.3.4, correcting each trait for age, sex, genotyping array and ancestry informative principal components 1 - 20 as previously described (14). GWAS involved high quality genome-wide imputed v3 genetic data (i.e., ~12 million SNPs, INFO > 0.9, MAF > 0.05%) measured in 448,010 related Europeans from the UK-Biobank Study that had both eBMD and height measured (15). GWAS meta-analysis summary results statistics for OA (Kindly made available by Arthritis Research UK Osteoarthritis Genetics (arcOGEN) Consortium) (16). Details of how eBMD and OA was defined and criteria for including individuals in each cohort are detailed in the original publications (15, 17, 18)

Gene-based tests of association encompassed a multi-model approach in which the association results from different gene analysis models were combined to produce an aggregate p-value corresponding to the strength of evidence of association between each protein coding gene (+2kb upstream / 1kb downstream) and eBMD, height, or OA. The two association models included: a SNP-wise mean $\chi2$ model [*i.e.* test statistic derived as the sum of −log(SNP p-value) for all SNPs that intersect the gene region of interest], and SNP-wise top $\chi2$ model [(test statistic derived as the sum of −log(SNP p-value) for top SNP in the region of interest)]. The aggregate approach was chosen as it yields a more even distribution of statistical power and sensitivity over a wider range of different genetic architectures. Importantly, due to incomplete genotyping coverage (i.e. no genetic variants were present in some gene), gene level associations could only be estimated for 465 (arcOGEN) - 482 (UK-Biobank Study) of the 483 DEGs, and 8,378 - 8,671/8,751 genes with background expression and 12,061 - 12,508/12,667 all mappable zebrafish – human orthologues. A reference sample of 50,000 unrelated European

individuals randomly selected from the UK Biobank Study was used to model patterns of linkage disequilibrium (LD) between variants.

Gene set analysis

Competitive gene set analysis was used to determine whether human orthologues of the 451 zebrafish DEG set was on average more strongly associated with adult height, eBMD or OA than all other human protein coding genes. The analysis accounted for several confounding factors including: gene size, gene density (i.e. representing the relative level of LD between SNPs in the gene) and the inverse of the mean minor allele count in the gene (i.e. to correct for potential power loss in very low minor allele count SNPs), as well the log value of these three factors. Because enrichment of the set of zebrafish DEGs was estimated relative to all human protein coding genes, we performed additional sensitivity analysis to ensure that our results were not biased by including human genes that (i) could not be confidently mapped between zebrafish and humans and (ii) that were not able to be assessed by RNA Seq as they did not pass QC (i.e.. these were not expressed in ontogenetic or regenerating scales). To achieve this, we generated two additional gene sets, one containing all mapped genes between zebrafish and humans (irrespective of whether they were expressed), and another containing the set of all genes that were mapped and expressed in ontogenetic and/or regenerating scales (i.e. background). We repeated the analysis, conditioning on both gene sets and thereby adjusted our baseline analysis for any "mapped genes vs all human gene effects" and / or "background genes vs all human gene effects".

Post hoc permutation analysis

Post hoc permutation analysis was performed using supplied R-scripts with default settings. A detailed description of the methods, in addition to interpretation of resulting plots is provided in the supplementary note from de Leeuw et al 2018 (19). Briefly, post-hoc permutation analysis involved generating quantile-quantile (QQ) plots that contrasted residual Z-scores (derived from gene-based tests of association above) of each human orthologue against its expected Z-score based on all of the genes in the set, using sample quantiles of the residual Z-scores. A one sided upper 95% confidence band was generated to monitor the degree to which the Z-score for each orthologue was likely to deviate from its expected Z-score using 10,000 permutations. Orthologues that exceeded the 95% confidence band were coloured in red and all other orthologues were coloured in grey. Plots were interpreted as follows: deviation from

the plot diagonal (i.e. expectation), suggested that the distribution of the observed values was different from the expected distribution. In cases where a gene set was not enriched, its residual Z-score distribution would match that of the data, and would tend to follow the plot diagonal, subject to some random variation. In contrast, if the gene set was enriched (and the association was attributable to phenotypic associations between all genes in the gene-set), the distribution of its residual Z-scores would differ from that of the data, as for every gene in the set. Consequently, the resulting distribution would deviate from the diagonal from the first gene onward. In the cases where enrichment is not due to all genes in the gene set, but rather a nested subset of genes, enrichment of the gene set would not be attributable to phenotype associations for all genes, and the resulting Z-score distribution would only differ from expectation for a subset of genes. Z-scores for the remaining genes would tend to be lower not deviate from expectation. The resulting mixture of distributions would result in a QQ-plot that, starting from the plot origin, would stay close to the diagonal initially, and deviate upwards later. The relative size of the overlap of genes in the gene set, with genes from the underlying pathway(s) that is enriched, would determine the deflection point, with smaller overlap resulting in a later (and often more pronounced) deflection.

Quantitative Real-Time PCR extended methods and primers

The cDNA qRT-PCR amplification reactions conditions were as follows: 3 min 95°C, 40 cycles of 15s 95°C and 1 min 60°C), ran on a CFX 96 (BioRad, Hercules, USA) qPCR machine, followed by a standard melting curve. Only amplicons passing the standard melting curve analysis were used for downstream calculations. The two reference genes (*eef1a1l1* and *rpl13*) and target amplicon primer sequences that have been used in this study can be found in the table below.

Primer sequences used in qPCR

Gene	Source	Forward primer (5’→3’)	Reverse primer (5’→3’)
acp5b	NM_001002452	CGTCCACTGACCACAGGAAGA	AAGGATCCTGACGTCTGATTGA
ctsk	NM_001017778.1	CTATAAAGAGATTCCTCAGGGTAACGA	ACACGGGTCCCACATTGG
bgna	NM_001002227.1	CCAACCTCTATGCCCTGGTC	TGGGCATGGATGTCAGAAGG
col10a1a	NM_001083827.1	GAAATGGGGGTTGGGGTTCA	TAAACCAACTCCAGGCGCTC
col11a2	NM_001079992.2	ATTGAAGAACAAGTCAAACCAAACC	GGCATGATCAGGAGTCCCAG
col1a2	AJ318213	GGAAACCTGAAGAAGGCTGTGT	TGAAAGTGAAGCGGCTGTTG
cx43	NM_131038.1	CACGCCGAAGGAACTGTCTA	GTCGAAGGCTTGTGCATGTG
eef1a1l1	NM_131263.1	CTGGAGGCCAGCTCAAACAT	TCAAGAAGAGTAGTACCGCTAGCATTAC
entpd5a	XM_679770.8	GCGGAACCCCAGATGGTT	CACCTGCAGCACCTCTTGGT
ihha	NM_001034993.2	GCTTGTGTACAGCGAGGTCA	CAAGCTCAGTTTCGCACCAG
mmp9	NM_213123.1	AAATCTGTGTTCGTGACGTTTCCT	GCCGTAACGCTTCAGATACTCAT
rpl13	NM_212784.1	TCTGGAGGACTGTAAGAGGTATGC	AGACGCACAATCTTGAGAGCAG
scpp5	NM_001145236.1	CATTCCCCACACAAGCGTTC	CTGAGCTCTACCGAAGTCGT
scpp7	NM_001145239.1	TATTGCGCTCCGCAAGTGAT	GCTGCCAAAAGAAGCTCGAT
sparc	AY575072.1	GAAGCCATTGAGGTCGTGGA	TGCCCTCCAGAGAGCATTTG
spp1	NM_001002308.1	GGCGGCTTGACATTTGTGAG	GTCCCCAACGGGAACAATCT
sp7	NM_212863.2	GGATACGCCGCTGGGTCTA	TCCTGACAATTCGGGCAATC

References (also cited in the main manuscript)

1. Dobin A, Davis CA, Schlesinger F, Drenkow J, Zaleski C, Jha S, et al. STAR: ultrafast universal RNA-seq aligner. Bioinformatics. 2013;29(1):15-21.
2. Love MI, Huber W, Anders S. Moderated estimation of fold change and dispersion for RNA-seq data with DESeq2. Genome Biology. 2014;15(12):550.
3. Sievers F, Wilm A, Dineen D, Gibson TJ, Karplus K, Li W, et al. Fast, scalable generation of high-quality protein multiple sequence alignments using Clustal Omega. Mol Syst Biol [Internet]. 2011 2011; 7:[539 p.].
4. The UniProt C. UniProt: a worldwide hub of protein knowledge. Nucleic Acids Research. 2018;47(D1):D506-D15.
5. Mudunuri U, Che A, Yi M, Stephens RM. bioDBnet: the biological database network. Bioinformatics. 2009;25(4):555-6.
6. Howe K, Clark MD, Torroja CF, Torrance J, Berthelot C, Muffato M, et al. The zebrafish reference genome sequence and its relationship to the human genome. Nature. 2013;496(7446):498-503.
7. Meyer A, Schartl M. Gene and genome duplications in vertebrates: the one-to-four (-to-eight in fish) rule and the evolution of novel gene functions. Current opinion in cell biology. 1999;11(6):699-704.
8. Mortier GR, Cohn DH, Cormier-Daire V, Hall C, Krakow D, Mundlos S, et al. Nosology and classification of genetic skeletal disorders: 2019 revision. American Journal of Medical Genetics Part A. 2019;179(12):2393-419.
9. Sudlow C, Gallacher J, Allen N, Beral V, Burton P, Danesh J, et al. UK biobank: an open access resource for identifying the causes of a wide range of complex diseases of middle and old age. PLoS medicine. 2015;12(3):e1001779.
10. de Leeuw CA, Mooij JM, Heskes T, Posthuma D. MAGMA: Generalized Gene-Set Analysis of GWAS Data. PLOS Computational Biology. 2015;11(4):e1004219.
11. Auton A, Abecasis GR, Altshuler DM, Durbin RM, Abecasis GR, Bentley DR, et al. A global reference for human genetic variation. Nature. 2015;526(7571):68-74.
12. Altshuler DM, Gibbs RA, Peltonen L, Altshuler DM, Gibbs RA, Peltonen L, et al. Integrating common and rare genetic variation in diverse human populations. Nature. 2010;467(7311):52-8.
13. Yang J, Lee SH, Goddard ME, Visscher PM. GCTA: a tool for genome-wide complex trait analysis. Am J Hum Genet. 2011;88(1):76-82.
14. Loh P-R, Tucker G, Bulik-Sullivan BK, Vilhjálmsson BJ, Finucane HK, Salem RM, et al. Efficient Bayesian mixed-model analysis increases association power in large cohorts. Nature Genetics. 2015;47(3):284-90.
15. Morris JA, Kemp JP, Youlten SE, Laurent L, Logan JG, Chai RC, et al. An atlas of genetic influences on osteoporosis in humans and mice. Nature Genetics. 2019;51(2):258-66.
16. Tachmazidou I, Hatzikotoulas K, Southam L, Esparza-Gordillo J, Haberland V, Zheng J, et al. Identification of new therapeutic targets for osteoarthritis through genome-wide analyses of UK Biobank data. Nature genetics. 2019;51(2):230-6.
17. Kemp JP, Morris JA, Medina-Gomez C, Forgetta V, Warrington NM, Youlten SE, et al. Identification of 153 new loci associated with heel bone mineral density and functional involvement of GPC6 in osteoporosis. Nat Genet. 2017.

18. Panoutsopoulou K, Southam L, Elliott KS, Wrayner N, Zhai G, Beazley C, et al. Insights into the genetic architecture of osteoarthritis from stage 1 of the arcOGEN study. Annals of the Rheumatic Diseases. 2011;70(5):864.
19. de Leeuw CA, Stringer S, Dekkers IA, Heskes T, Posthuma D. Conditional and interaction gene-set analysis reveals novel functional pathways for blood pressure. Nature Communications. 2018;9(1):3768.
